# A model for regional‐scale oak savanna management: The roles of fire, canopy, and soils for understory plant diversity

**DOI:** 10.1002/eap.70120

**Published:** 2025-10-15

**Authors:** Tyler J. Bassett, Eric Behrens, Ralph Grundel, Johanna Nifosi, Noel B. Pavlovic, Lars A. Brudvig

**Affiliations:** ^1^ Department of Plant Biology Michigan State University East Lansing Michigan USA; ^2^ Michigan Natural Features Inventory Michigan State University Extension Lansing Michigan USA; ^3^ National Park Service, Southern Plains/Rio Grande Fire Groups, Lake Meredith National Recreation Area Fritch Texas USA; ^4^ U.S. Geological Survey, Great Lakes Science Center, Lake Michigan Ecological Research Station Chesterton Indiana USA; ^5^ Program in Ecology, Evolution, and Behavior Michigan State University East Lansing Michigan USA

**Keywords:** context‐dependent, management, oak savanna, plant diversity, prescribed fire

## Abstract

Predicting the outcomes of land management on biodiversity is difficult without a mechanistic understanding of how management approaches, ecosystem structure, environmental conditions, and biodiversity interact. Management effects may be direct or indirect, context‐ or scale‐dependent, or obscured by local environmental conditions. Resolving these relationships at the regional scale may be difficult, given heterogeneity in local environmental conditions, yet understanding broad‐scale patterns can elucidate context dependencies and improve restoration outcomes. We confronted these challenges within globally rare oak savannas in the midwestern United States, which have been altered by fire exclusion and resulting woody encroachment. By modeling direct and indirect pathways by which management influences diversity, we test a general framework for savanna restoration. Across 100 oak savannas spanning five US states, management by prescribed fire and mechanical thinning of woody vegetation affected groundlayer plant species richness through changes to ecosystem structure (canopy openness and litter depth), and these effects were both context‐ and scale‐dependent. Frequent prescribed fires and canopy thinning promoted greater canopy openness, which in turn increased richness at small (1 m^2^), but not larger (1000 m^2^) scales. Frequent fire additionally increased richness at small and larger scales through effects independent of ecosystem structure. While management effects were large relative to the influence of local edaphic conditions, soil productivity had two largely offsetting effects on small‐scale richness, increasing richness directly but decreasing richness indirectly by promoting closed canopy structure. These results suggest using a combination of fire and canopy thinning to reverse the effects of decades of fire exclusion. However, management effects were also context‐dependent, emphasizing that management outcomes vary regionally. Here, 1‐m^2^ plant species richness increased with both fire frequency and canopy thinning under low, but not high, productivity soil conditions. By demonstrating how specific management practices influence savanna structure and biodiversity by manipulating ecological processes across broad geographic and edaphic gradients, our findings provide a framework for understanding management outcomes at short and medium intervals (e.g., within and between decades, respectively), in the form of a model that can be refined by testing additional hypotheses to better predict savanna restoration outcomes.

## INTRODUCTION

Grasslands and savannas are widespread globally and important culturally, economically, and for the biodiversity they support (Strömberg & Staver, 2022; Trothill & Mott, 1985). They are also among the most imperiled ecosystems on Earth, due to extensive land‐use conversion and altered disturbance regimes (Buisson et al., 2022; Carbutt et al., 2017; Hoekstra et al., 2005). In the absence of regularly occurring disturbances such as fire, grazing, and drought, encroachment by woody vegetation can convert open grasslands and savannas to shrublands, woodlands, or forests with concomitant biodiversity losses (Bond, 2021; Grundel & Pavlovic, 2007; Lehmann et al., 2014; Murphy & Bowman, 2012; Ratajczak et al., 2012; Stevens et al., 2017). Thus, grassland and savanna restoration depends on the reinstatement of historic disturbance regimes to reverse or prevent woody encroachment (Buisson et al., 2022; McPherson, 1997). However, the extent of changes to ecosystem structure (e.g., woody encroachment) in grasslands and savannas that have succeeded to shrublands, woodlands, and forests may be great enough that reinstating historical disturbance regimes alone is insufficient to restore historical structure and biodiversity, and additional strategies to reverse woody encroachment are required (Bond, 2021; Suding et al., 2004).

We address the degree to which historic disturbance regimes can recover structure and groundlayer plant biodiversity within midwestern North America oak savannas. Midwestern oak savannas occur between the prairies of the Great Plains and the Eastern Deciduous Forest biome (Anderson et al., 1999; McPherson, 1997; Nuzzo, 1986). When maintained by frequent surface fires (3–5 fires/decade; Peterson & Reich, 2008; Wolf, 2004), midwestern oak savannas are characterized by a scattered canopy of primarily oak (*Quercus* L. species) trees. The resulting heterogeneous understory light environment can support a diverse and densely vegetated groundlayer (Asbjornsen et al., 2005; Leach & Givnish, 1999; Pavlovic et al., 2006). Without frequent surface fires, midwestern oak savannas are invaded by fire‐sensitive trees and shrubs and convert to closed‐canopied forests, with resulting declines in groundlayer plant diversity (Briggs et al., 2005; Brudvig & Mabry, 2008; Ladwig et al., 2018; Nowacki & Abrams, 2008; Rogers et al., 2008). Despite significant research on the role of fire in savannas in individual sites or narrow regions (Tester, 1996; Yantes et al., 2023), we still lack a framework to predict the restoration of groundlayer plant diversity across broad geographic, edaphic, and management history gradients in the midwestern United States (Asbjornsen et al., 2005; Yantes et al., 2023).

Articulating such a framework requires resolving several challenges. First, managers use multiple restoration methods, including fire and mechanical thinning, but the efficacy of each alone or in combination is unclear. Prescribed fire is a fundamental tool in oak savanna restoration (Spencer et al., 2017). However, across a range of canopy closure and woody encroachment, it remains unclear whether, or over what timeline, prescribed fire alone will reverse these structural changes and restore groundlayer diversity. In some instances, frequent use of prescribed fire can reduce woody encroachment and restore characteristic structure and composition, especially when applied over long time periods (every 1–3 years over 30–60 years; Abella et al., 2023; Knapp et al., 2015; Peterson & Reich, 2001, 2008). In other cases, however, prescribed fire alone has been insufficient as a restoration tool, or has yielded ephemeral results (Bassett et al., 2020; Bowles et al., 2017). Low fire intensity due to insufficient fuels, a high density of fire‐resistant trees, and rapid infilling and resprouting of woody species following fire can prevent fire from effectively reducing woody vegetation density (Abella et al., 2004; Davis, 2021; Knapp et al., 2022; Nowacki & Abrams, 2008). Successful restoration may require that fire and mechanical thinning of shrubs and trees be used together to reduce woody encroachment (Bassett et al., 2020; Bowles et al., 2017; Nielsen et al., 2003; Peterson & Reich, 2008; Yantes et al., 2023). Finally, mechanical thinning by itself may promote open canopies but lacks the added benefits of fire for stimulating groundlayer plant diversity such as promoting soil nutrient pulses and exposing bare ground microsites for seedling recruitment (Brudvig, 2010; Brudvig & Asbjornsen, 2009; Tester, 1996).

Second, the efficacy of managing with fire or thinning may be context‐dependent, overwhelmed or modified by underlying environmental conditions (Brudvig, 2011; Grman et al., 2013). Resolving these context dependencies is particularly important over the large geographic extent that midwestern oak savannas cover, which spans substantial variation in local environmental conditions, land‐use and management history, landscape context, and species pools (Anderson et al., 1999). For example, on less productive soils, fire may promote open canopy conditions more effectively due to drier fuel conditions resulting in higher severity fires, greater rates of tree mortality during fire, and slower overall rates of canopy infilling (Leach & Givnish, 1999).

Third, management approaches and environmental conditions can affect the restoration of groundlayer plant diversity directly, or indirectly by modifying each other. Light and soil resource availability is important for driving groundlayer diversity, including in oak savannas (Leach & Givnish, 1999). Both frequent fire and mechanical thinning can increase understory light availability by reducing tree canopy cover, increasing groundlayer plant diversity and driving shifts in composition (Brudvig, 2010; Leach & Givnish, 1999; Peterson et al., 2007; Weiher, 2003). The influence of soil resources on diversity may be independent of management effects, or management may influence diversity via effects on soil resources, for example if fire accelerates nutrient cycling leading to pulses in soil resource availability (Leach & Givnish, 1999; Meisel et al., 2002).

Finally, management and environmental conditions can have scale‐dependent effects within oak savannas, so interpreting restoration outcomes may require conducting assessments at multiple scales, especially if environmental and management effects operate via different mechanisms at different scales (Catano et al., 2021; Sluis et al., 2018). For example, fire might promote small scale diversity (e.g., 1‐m^2^) by opening microsites for seedling recruitment (Myers & Harms, 2011) and larger scale diversity by preventing site‐level extinctions (Pavlovic et al., 2011).

We addressed the challenge of understanding relationships among management, environmental conditions, and scale through a regional‐scale assessment of how midwestern oak savanna management affects ecosystem structure and groundlayer plant diversity. Our study spanned 100 sites and five states across the US Great Lakes region (Illinois, Indiana, Ohio, Michigan, and Wisconsin) and considered multiple management approaches: prescribed fire alone; prescribed fire with mechanical thinning of shrubs, saplings, and canopy trees; mechanical thinning alone; and no recent management. We evaluated the relative importance of several hypothesized drivers of groundlayer plant species richness in midwestern oak savannas to address how management influences richness directly and indirectly through changes to ecosystem structure and how the effects of management vary across environmental context and spatial scale. We combined these processes in a conceptual and modeling framework that comprises a fundamental hypothesis linking management to multiple pathways that predict the restoration of groundlayer plant diversity in midwestern oak savannas. Specifically, we asked:

1. How do management (fire, canopy thinning, shrub, and sapling thinning) and soil productivity influence groundlayer plant species richness, and to what extent are these relationships mediated through changes to ecosystem structure (canopy openness, litter depth)? We predicted that management with prescribed fire and mechanical thinning would increase groundlayer plant diversity by increasing canopy openness and that fire would additionally increase groundlayer plant diversity by reducing leaf litter depth. We predicted that groundlayer diversity would increase with soil productivity.
2. How does the relationship between management and species richness, and management and ecosystem structure, vary across a soil productivity gradient? We predicted stronger effects of fire frequency on both plant diversity and ecosystem structure with decreasing soil productivity.
3. To what extent are the answers to questions 1 and 2 scale‐dependent? We predicted that direct and indirect management effects and the effects of environmental conditions (e.g., soil productivity) and small‐scale structural factors (e.g., litter depth) on groundlayer plant diversity would be stronger at small scales because the factors that determine plant community composition (e.g., soil productivity, light) often vary at small scales (Weiher, 2003).

## METHODS

### Survey area

**FIGURE 1:**
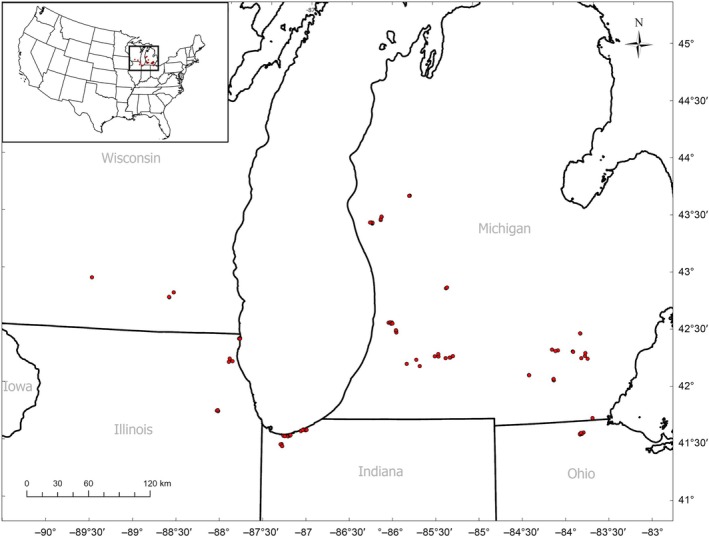
Map of the 100 oak savanna study locations, spanning the Great Lakes region of Ohio, Illinois, Indiana, Michigan, and Wisconsin. Inset showing location within the conterminous United States.

We assessed how variation in management history in oak savannas affected groundlayer plant communities by sampling 100 sites within the US Great Lakes region of Illinois, Indiana, Ohio, Michigan, and Wisconsin. Our study area ranged from Toledo, Ohio, in the southeast (41.619° N, −83.792° W), to Indiana Dunes National Park, Indiana, in the southwest (41.634° N, −87.054° W), to Madison, Wisconsin, in the northwest (42.945° N, −89.589° W), and to the Manistee National Forest, Michigan, in the north (43.720° N, −85.797° W) (Figure 1; Appendix S1: Table S1). Average annual precipitation and temperatures range from 87.6 to 108.5 cm and from 11.4 to 15.4°C, respectively (NWS, 2024).

### Study design

We sampled sites that historically supported oak savannas (Bowles et al., 2015; Comer et al., 1995; Finley, 1951; Gordon & Flint, 1966; Illinois Department of Natural Resources, Illinois Natural History Survey, 2003), are currently undergoing restoration for oak savanna using a variety of management approaches, and that represent a range of soil resource conditions. We identified geographically proximal clusters of two to seven edaphically similar sites that represented a variety of management histories, differing in the frequency and intensity of canopy thinning, shrub and sapling thinning, and prescribed fire (Appendix S1: Table S1). Most clusters included at least one site with each of the following histories—one or more prescribed fires, mechanical canopy or shrub and sapling thinning, a combination of burning and thinning, and no management with fire or thinning in the 40 years prior to sampling. Our goal in identifying a range of management histories within clusters was to control for measured (e.g., soil characteristics) and unmeasured (e.g., land‐use history) environmental gradients, while testing for differences among management approaches.

Sampling occurred from mid‐July to mid‐September in 2017–2019. We established a randomly oriented 20‐m × 50‐m (0.1‐ha) plot, placed in the approximate center of each site through visual estimation, and oriented perpendicular to the slope to encompass the greatest variation in site conditions. We collected data on plant species composition and abundance, as well as structural components hypothesized to affect groundlayer plant community composition that are the target of management actions, including leaf litter depth and canopy openness (Dey et al., 2017; Appendix S1).

We collected data on vascular plant species composition and abundance at three vertical strata, the ground, shrub, and tree layers. Along a 45‐m transect through the center of the plot, we recorded data on groundlayer vascular plant composition and abundance in 10, 1‐m^2^ quadrats at 5‐m intervals along the transect. We identified each vascular plant species in the groundlayer (all herbaceous vegetation and woody species under 50 cm tall) and estimated the percent cover of each taxon. We estimated cover, one species at a time, whereby each species could cover up to 100% of the plot and the cumulative percent cover of plant species could exceed 100% in dense quadrats where species overtopped each other. After sampling quadrats, we conducted a meander survey of the full 0.1‐ha plot and recorded the presence of any vascular plant species that were not recorded in the quadrats. We recorded the species and counted the number of stems of each shrub and sapling (woody species < 5 cm dbh; breast height = 1.4 m) in a 2‐m belt transect centered on the 45‐m transect. Finally, we recorded the species and dbh of each tree (woody species ≥ 5 cm dbh) in the 0.1‐ha plot. When unable to identify plants to species, we identified plants to genus and lumped all unidentifiable species within that genus together for calculations of richness. Plant nomenclature followed Voss and Reznicek (2012).

We recorded data on ecosystem structure in the 1‐m^2^ quadrats along each 45‐m transect, specifically litter depth and canopy cover. We measured litter depth to the nearest 0.5 cm at each of the four corners of every quadrat and calculated the mean of these four values prior to analysis (Appendix S1: Figure S1a). We recorded four canopy openness measurements (one in each cardinal direction) every 5 m along the transect, above each quadrat, with a densiometer, and calculated the mean of these four values prior to analysis (Appendix S1: Figure S1b). Finally, we collected a 20‐cm‐deep by 3‐cm^2^ soil core at the midpoint of each side of each quadrat, pooling all 40 soil cores for each transect, subsequently analyzing the pooled sample for soil organic matter, soil texture (sand, silt, and clay content), Mehlich‐III phosphorus, Bray‐II phosphorus, % nitrogen, total ion exchange capacity, and pH (Brookside Laboratories, New Knoxville, Ohio, USA) (Appendix S1: Figure S1c). Separately, we assessed soil water‐holding capacity by calculating the proportion of oven‐dried weight to saturated wet weight and used site means for analysis (Appendix S1: Figure S1d; Brudvig & Damschen, 2011).

We assembled data on management history by collecting field data and conducting interviews with land managers. Each land manager reported the date of each shrub and sapling thinning, canopy thinning, and prescribed fire, dating to when record‐keeping began at each site. To estimate the intensity of shrub and sapling thinning, we counted the number of woody stems <5 cm diameter that were cut under breast height in the shrub belt transect, which could include any tree sapling or shrub that had previously attained at least breast height. Shrub and sapling thinning was achieved using either hand tools or a forestry mower, and with or without stump‐treatment with herbicide. To estimate the intensity of canopy thinning, we measured the diameter of each woody stem ≥5 cm diameter that was cut under breast height in the full plot. We also separately counted and measured stems that were downed due to windfall. We reasoned that the pre‐management stem density could be approximated by summing data for cut and live stems (refer to the following under *Data analysis*).

### Data analysis

We conducted analyses in R v.4.0.3 (R Core Team, 2020).

We calculated plant species richness at two scales, both the 1‐m^2^ quadrat‐level mean across all 10 quadrats along the transect (species richness/1 m^2^) and the total species richness in the entire 0.1‐ha plot, including 1‐m^2^ quadrats (species richness/1000 m^2^) (Appendix S1: Figure S1e). Across sites, the proportion of species richness/1 m^2^ composed of native species ranged from 0.56 to 1.00 (mean = 0.92, median = 0.96). The proportion of species richness/1000 m^2^ composed of native species ranged from 0.67 to 1.00 (mean = 0.87, median = 0.89).

We used principal components analysis (PCA) (Greenacre et al., 2022) to create one variable describing soil conditions, due to correlations among soil variables. The soil PCA included soil organic matter, soil texture (percent sand, silt, and clay content), Mehlich‐III phosphorus, Bray‐II phosphorus, pH, total ion exchange capacity, and water‐holding capacity (in percentage). The first PC axis described 60% of the variation in soil variables and was positively related to percent silt, percent clay, soil organic matter, total exchange capacity, and water‐holding capacity and negatively related to percent sand (Appendix S1: Figure S2, Table S2).

We calculated shrub and sapling thinning intensity as number of cut shrub and sapling stems/(number of cut shrub and sapling stems + number of live shrub and sapling stems), and canopy thinning intensity as total dbh of cut stumps/(total dbh of cut stumps + total dbh of standing trees). To describe fire frequency, we calculated the number of burns since the year 2000 (Appendix S1: Figure S1f). We used the year 2000 as a reference point because fire records prior to 2000 were not consistently known for many sites, and we assumed that the effects of older fires were less closely linked to the responses we measured.

### Question 1: How do management and soil influence richness directly and via structure?

We constructed and analyzed structural equation models (SEMs) in the *Lavaan* package in R (Rosseel, 2012) to explore how management history and soil productivity influence ecosystem structure and groundlayer species richness, and whether the effect of management and soil on richness is mediated through structure. We constructed two models, one each for species richness/1 m^2^ and species richness/1000 m^2^. First, we constructed a meta‐model of hypothesized relationships among our study variables (Appendix S2: Figure S1). Each path included in the model was based on a hypothesized causal relationship expected from theory or previous observation (Appendix S2). We then calculated modification indices, which indicate the degree to which each unused path or correlation would improve the fit of the model (e.g., reduce χ^2^ value), and considered adding paths with modification indices >3.84 (equivalent to a parameter having a *p* < 0.05). All path coefficients were standardized, so their values represent relative effect sizes.

To complement the SEMs, we constructed a separate linear model for each endogenous variable (canopy openness, litter depth, species richness/1 m^2^, and species richness/1000 m^2^). We present partial regression plots for each bivariate relationship, controlling for the causal (independent) variables predicting each endogenous (dependent) variable in the SEM analyses. Because partial bivariate relationships from linear models do not account for the covariance structure of SEMs, we use them to help visualize and interpret relationships within the SEMs but rely on the SEMs for our main results.

### Question 2: How do management effects vary across a soil productivity gradient?

To address question 2, whether the effects of management on ecosystem structure and groundlayer plant species richness vary along the soil gradient or with management intensity, we constructed linear mixed models with cluster as a random effect and year of sampling as a fixed effect, using the *lmer* function in the *lme4* package in R (Bates et al., 2015). We tested four models, one each for canopy openness, litter depth, species richness/1 m^2^, and species richness/1000 m^2^. To test for variation in management effects across a soil productivity gradient, we included terms for the interaction between soil productivity and each management strategy—canopy thinning, shrub and sapling thinning, and fire frequency. We included terms for the main effects of all four factors. All variance inflation factors were <2.5. We initially included the duration of management as an additional factor, but duration and intensity of management were highly correlated. For example, sites with a longer fire history were also burned more times since 2000 (Pearson's correlation; *r* = 0.50, *p* < 0.001), and their interaction increased multicollinearity in models (variance inflation factor > 6).

### Question 3: Are effects on richness scale‐dependent?

We addressed questions of scale‐dependency by conducting the analyses used to address Questions 1 and 2 using both species richness/1 m^2^ and species richness/1000 m^2^ as response variables. Statistical tests were significant if *p* < 0.05.

## RESULTS

### Question 1: How do management and soil influence richness directly and via structure?

**FIGURE 2:**
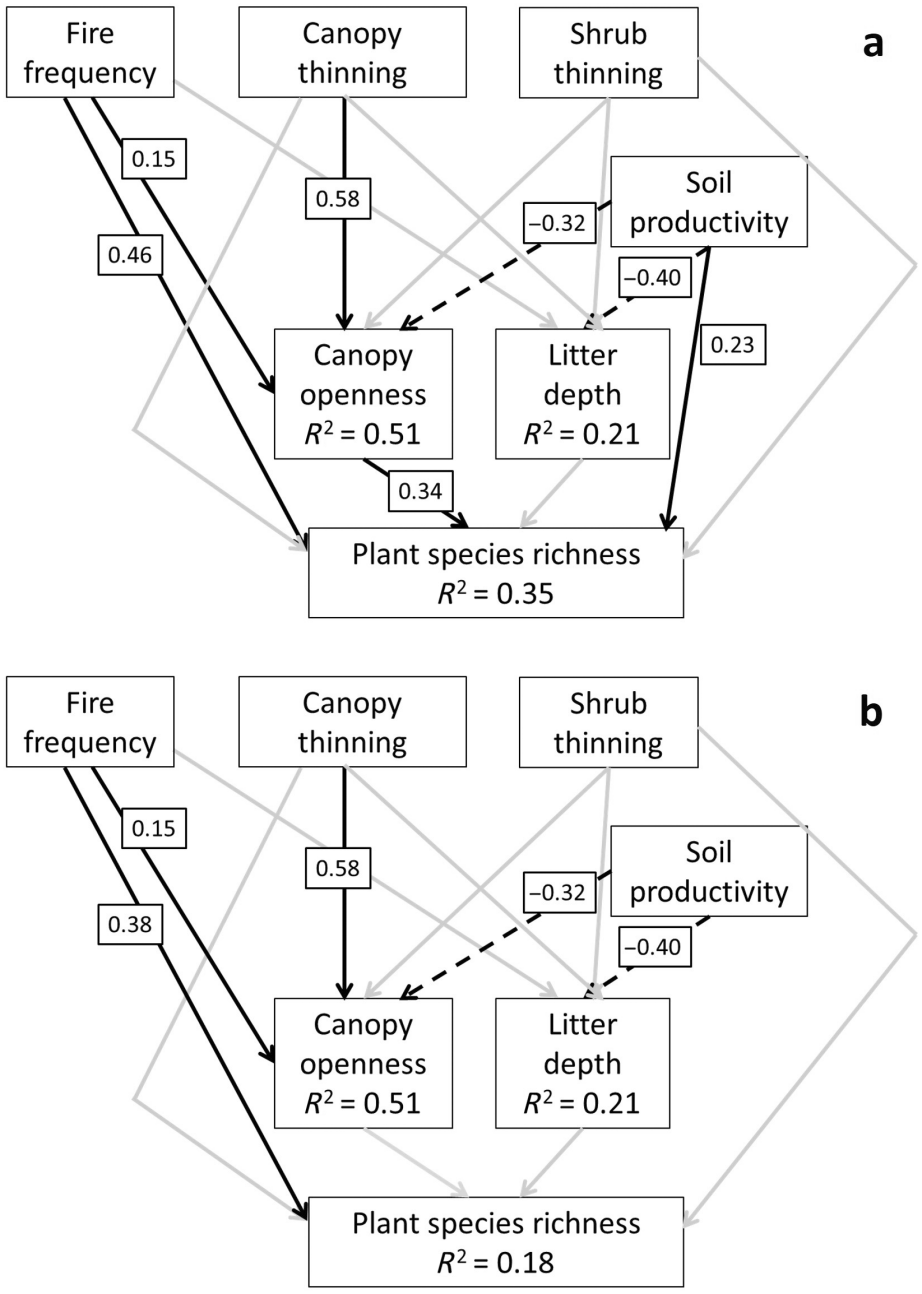
Final structural equation models for 1‐m^2^ richness (a) and 1000‐m^2^ richness (b). Statistically significant effects (*p* < 0.05) are indicated by black lines, all others (*p* > 0.05) are indicated by gray lines. Path coefficients are displayed in small boxes for all statistically significant effects. Positive effects are indicated by solid lines, and negative effects are indicated by dashed lines. Proportion of variance explained (*R* ^2^) is given for each endogenous variable.

We found that both management history and soil productivity influenced ecosystem structure and groundlayer species richness, and that the effect of management on richness was partially mediated through structure, while the effect of soil was not. An initial SEM without a direct path from soil productivity to plant species richness fit poorly for the species richness/1 m^2^ model (*p* = 0.04, χ^2^ = 6.48). A modification index (m.i. = 4.6) indicated inclusion of a path from soil productivity to 1‐m^2^ richness and a model including that path fit well (*p* = 0.21, χ^2^ = 1.59). A species richness/1000‐m^2^ richness model without a direct path from soil productivity to plant species richness fit well (*p* = 0.40, χ^2^ = 1.89). No modification indices for the 1000‐m^2^ richness model indicated that additional paths would improve fit (including for a path from soil productivity to 1000‐m^2^ richness, m.i. = 0.25), so we did not fit a new model. The final 1‐m^2^ richness SEM explained between 21% and 52% of the variation in litter depth (*R* ^2^ = 0.21), canopy openness (*R* ^2^ = 0.51), and plant species richness (*R* ^2^ = 0.35) (Figure 2a). The final 1000‐m^2^ richness SEM explained the same variation in litter depth and canopy openness and less variation in plant species richness (*R* ^2^ = 0.18) (Figure 2b).

**FIGURE 3:**
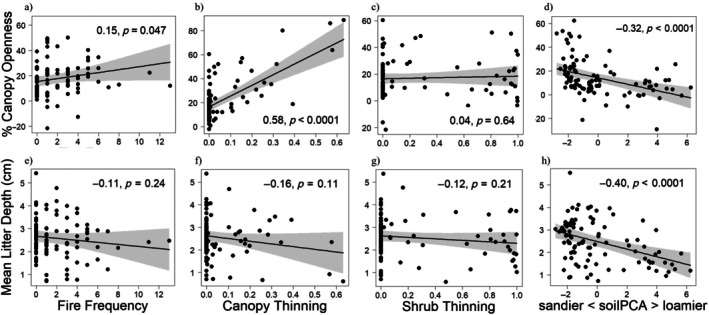
Bivariate relationships with 95% CIs of individual paths predicting percent canopy openness (top row) and litter depth (bottom row) with, respectively, fire frequency (a and e), canopy thinning (b and f), shrub thinning (c and g), and soil principal components analysis (PCA) (d and h) in the oak savanna structural equation models (SEMs). Partial‐residual plots are from linear regressions including all predictors shown. *Y*‐axes represent modeled values when controlling for other variables, so may be outside of the measured range. *R* ^2^ coefficients and *p* values are from the corresponding path in the 1‐m^2^ and 1000‐m^2^ SEM models. Fire frequency = number of burns since 2000; canopy and shrub thinning = proportion of basal area and stems removed, respectively.

In both the 1‐m^2^ and 1000‐m^2^ richness models, ecosystem structure was related to management and soils (Figure 2). Canopy openness increased with the intensity of canopy thinning and with fire frequency, but not with shrub and sapling thinning intensity. The effect size of canopy thinning on canopy openness was nearly four times that of fire frequency (*r* = 0.58, *p* > 0.001 vs. *r* = 0.15, *p* < 0.05; Figure 3). Canopy openness also decreased with soil productivity (*r* = −0.32, *p* < 0.001; Figure 3). Leaf litter depth increased with soil productivity (*r* = 0.40, *p* < 0.001), but was not significantly associated with fire frequency, canopy thinning, or shrub and sapling thinning (Figure 3).

**FIGURE 4:**
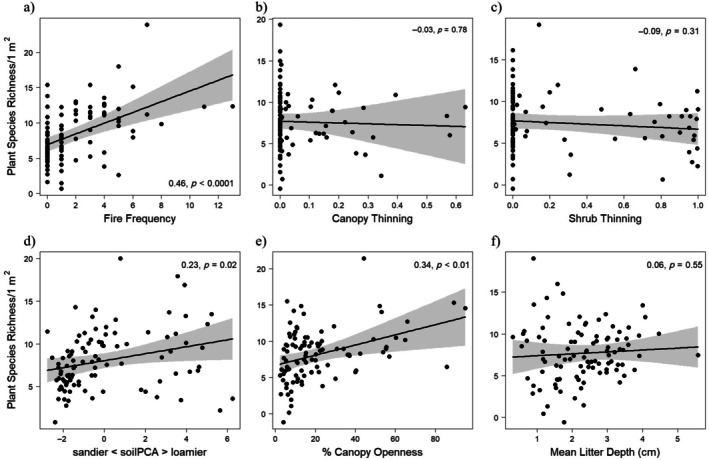
Bivariate relationships with 95% CIs of individual paths predicting species richness/1 m^2^ in structural equation models (SEMs) (Figure 3) from 100 oak savanna sites across the lower Great Lakes region. Partial‐residual plots are from linear regressions including (a) fire frequency, (b) canopy thinning, (c) shrub thinning, (d) soil principal components analysis (PCA) gradient, (e) canopy openness, and (f) mean litter depth. *R* ^2^ coefficients and *p* values are from the corresponding path in the 1‐m^2^ SEM model. Fire frequency = number of burns since 2000; canopy and shrub thinning = proportion of basal area and stems removed, respectively.

Plant species richness at the 1‐m^2^ scale increased with fire frequency, both directly (*r* = 0.46, *p* < 0.001) and indirectly via increased canopy openness (total standardized effect size [TSE], 0.15 × 0.34 = 0.05) (Figures 2a and 4). Plant species richness at the 1‐m^2^ scale increased with canopy thinning indirectly via increased canopy openness (TSE, 0.58 × 0.34 = 0.20). Plant species richness at the 1‐m^2^ scale increased with soil productivity directly (0.23, *p* < 0.05; Figure 4), but decreased with soil productivity indirectly via decreased canopy openness (TSE, −0.32 × 0.34 = −0.11). Litter depth was not significantly correlated with plant species richness (Figure 4), and soil productivity did not affect plant species richness indirectly through its correlation with litter depth.

**FIGURE 5:**
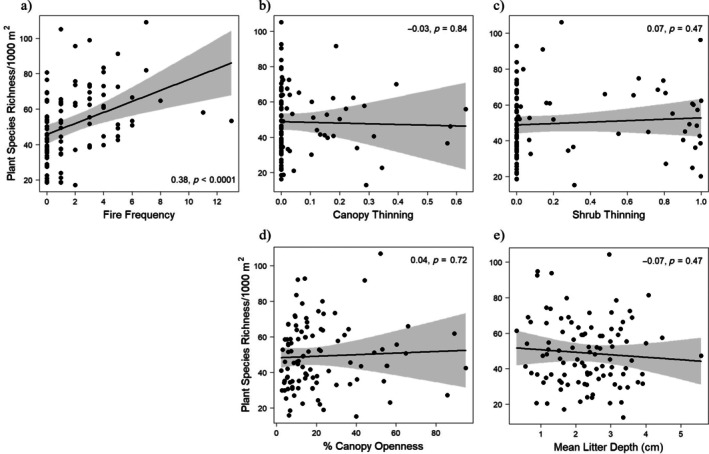
Bivariate relationships with 95% CIs of individual paths predicting species richness/1000 m^2^ in structural equation models (SEMs) for models based on data from 100 oak savanna sites (Figure 4). Graphs are species richness per 1000 m^2^ with (a) fire frequency, (b) canopy thinning, (c) shrub thinning, (d) canopy openness, and (e) mean litter depth. Partial‐residual plots are from linear regressions including all predictors shown. Coefficients and *p* values are from the corresponding path in the 1000‐m^2^ SEM model. Fire frequency = number of burns since 2000; canopy and shrub thinning = proportion of basal area and stems removed, respectively.

Plant species richness at the 1000‐m^2^ scale increased directly with fire frequency (*r* = 0.38, *p* < 0.001), but was not significantly associated with any other model parameters (Figures 2b and 5). No indirect effects were statistically significant on 1000‐m^2^ richness (Figure 2b).

### Question 2: How do management effects vary across a soil productivity gradient?

**FIGURE 6:**
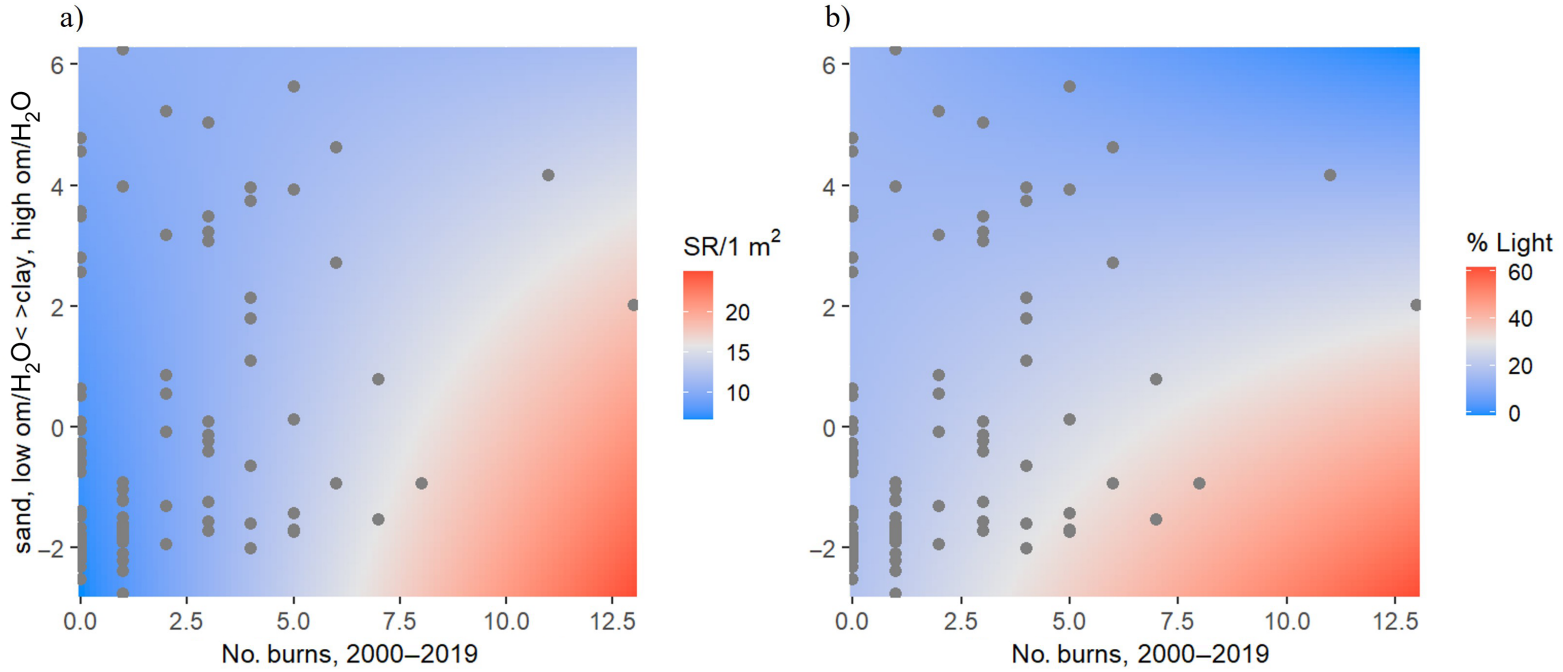
1‐m^2^ richness (species richness [SR]/1 m^2^) (a) and canopy openness (% Light) (b) increased with fire frequency (burns, 2000–2019) at less productive sites (negative soil PC1 values: sand, low organic matter [om], and water‐holding capacity [H_2_O]), but not in more productive sites (positive soil PC1 values). PC1, first principal component.

The effect of management on canopy openness and groundlayer species richness varied across the soil productivity gradient (Table 1). There was an interaction between fire frequency and canopy openness (*p* < 0.05), whereby fire frequency and canopy openness were positively related at sites with unproductive soils (negative values on soil PC1), but there was little relationship between fire frequency and canopy openness at sites with productive soils (Figure 6b). Similarly, there was an interaction between canopy thinning and soil productivity for canopy openness (*p* < 0.01). Canopy thinning and canopy openness were positively correlated under unproductive conditions, but not under productive conditions, although this interactive effect should be interpreted with caution as canopy thinning intensity was skewed toward low productivity sites (Appendix S3: Figure S1). There was also an interaction between shrub and sapling thinning and soil productivity for canopy openness (*p* < 0.05), with a positive relationship between shrub and sapling thinning and canopy openness only under unproductive soil conditions (Appendix S3: Figure S2). There were also interactions between fire frequency and soil productivity for 1‐m^2^ species richness and canopy thinning and soil productivity for 1000‐m^2^ richness (Table 1). Fire frequency positively correlated with 1‐m^2^ species richness under low soil productivity, but not under high soil productivity (Figure 6a). Canopy thinning was positively correlated with 1000‐m^2^ species richness under high soil productivity, but not under low soil productivity (Appendix S3: Figure S3). No other interactive effects were statistically significant (Table 1). Example results are also presented pictorially in Appendix S4, demonstrating different treatment effects (fire frequency, canopy thinning, shrub and sapling thinning) at different soil productivity levels.

**TABLE 1:** Management effects across soil productivity gradient, showing coefficients for fixed effects from generalized linear mixed model with cluster as a random factor and controlling for year as a fixed factor (^^^ *p* < 0.10, *p < 0.05, **p < 0.01, ***p < 0.001).

	Fire frequency	Canopy thinning	Shrub thinning	Soil PCA	Fire × soil	Canopy × soil	Shrub × soil	Marginal‐*R* ^2^
Canopy openness	1.94**	65.19***	0.99	−0.54	−0.49*	−19.78**	−3.34*	0.57
Litter depth	−0.06	−0.60	−0.44^^^	−0.28***	0.02	0.37	0.10	0.28
SR/1 m^2^	1.02***	6.99*	−1.02	0.41	−0.14*	2.18	0.08	0.34
SR/1000 m^2^	3.73***	22.26	3.54	0.90	−0.56	17.78*	−0.08	0.21

*Note*: Coefficients and significant designations are bolded if statistically significant. Marginal *R* ^2^ is the proportion of variance explained by the fixed effects relative to the overall variance. Canopy openness (in percentage) was measured with a densiometer, litter depth (in millimeters) from mineral soil to top of the litter, mean species richness (SR) of the ten 1‐m^2^ plots yielded SR/1 m^2^, and total species richness for meter squared plots plus new species from meander survey yielded SR/1000 m^2^. Fire frequency = number of burns since 2000; canopy and shrub thinning = proportion of basal area and stems removed, respectively. The soil gradient is derived from a principal components analysis (PCA) of soil organic matter, soil texture (percent sand, silt, and clay content), Mehlich‐III phosphorus, Bray‐II phosphorus, pH, total ion exchange capacity, and water‐holding capacity. “×” indicates an interaction between factors.

## DISCUSSION

The predictability of oak savanna management outcomes can be improved if management is guided by a generalizable framework describing how management approaches, ecosystem structure, and biodiversity relate to one another. Here, we present such a framework and test for the relative strength of key relationships in a modeling approach that incorporates both direct effects of management on groundlayer plant diversity and indirect effects via manipulations of ecosystem structure. Through the study of 100 oak savannas spanning five states, we found that more intense canopy tree thinning and more frequent prescribed fire increased groundlayer plant species richness through effects on ecosystem structure (canopy openness but not litter depth). These effects, however, were both scale‐ and context‐dependent, with influences evident at 1 m^2^ that were absent at the 1000‐m^2^ sampling scales, and the effects of management were modified by underlying soil conditions. Together, these findings point to an important influence of ecosystem management for oak savanna biodiversity patterns and contribute to a generalized approach for predicting management outcomes at a broad regional scale.

### Drivers of groundlayer plant diversity and the influences of management practices

Our findings point to understory light availability as a key driver of groundlayer plant diversity in oak savannas across our study region. By promoting canopy openness, frequent prescribed burning and canopy tree thinning increase groundlayer light levels in this ecosystem (Lettow et al., 2014). By demonstrating the geographic breadth of this pattern, our study emphasizes the importance of numerous studies that have explored the mechanisms behind this pattern locally (Abella et al., 2023; Bassett et al., 2020; Brudvig, 2010; Leach & Givnish, 1999; Noble & Bauer, 2022; Peterson et al., 2007). For example, Noble and Bauer (2022) found that groundlayer richness was highest due to greater forb and legume diversity at intermediate canopy cover in the Indiana Dunes National Park, Indiana. In Ohio, Abella et al. (2023) identified thresholds in canopy cover (<200 trees/ha, basal area < 10 m^2^/ha) that maximized oak savanna groundlayer richness, in addition to frequent and recent fire (>1 fire in the last 3 years). Importantly, we modeled the relative importance of different management factors for increasing richness via light availability and found that the effect of canopy tree thinning on canopy openness was four times stronger than that of prescribed burning (TSE = 0.19 vs. 0.05; Figure 2a). Multiple fires over decades are required to build up sufficient fuels to induce canopy mortality, especially when compared to the rapid effects of tree thinning on canopy openness (Brudvig & Asbjornsen, 2009; Tester, 1996). The extent of encroachment by fire‐resistant woody species at our study sites likely reduced fire severity and limited canopy tree mortality. Prior to European colonization, Indigenous cultures set frequent landscape‐scale fires that maintained open‐canopied conditions in midwestern oak savannas, but reversing up to a century or more of fire exclusion requires repeated prescribed fires over decades to open oak savanna canopies through mortality of fire‐sensitive tree species (Chapman & Brewer, 2008; Cottam, 1949; Knapp et al., 2015; Nowacki & Abrams, 2008; Peterson & Reich, 2001).

Our findings indicate that frequent prescribed burning also promoted plant species richness independently of changes to ecosystem structure at both 1 m^2^ and 1000 m^2^ spatial scales. These effects were large relative to the influences of fire on richness that were mediated through canopy openness. The TSE for the direct effect of fire on 1‐m^2^ richness was nine times greater than the indirect effect of fire mediated by canopy openness (TSE = 0.46 vs. 0.05). Multiple mechanisms may have underpinned these direct fire effects, including greater rates of flowering and seed production by groundlayer plant populations at sites managed with frequent fire, or more frequent exposure of bare ground as microsites for recruitment (Myers & Harms, 2011; Pavlovic et al., 2011). Additional work could aid in resolving the specific mechanisms for this direct influence. Regardless, our study indicates that frequent prescribed fire operated through multiple mechanisms to be a major driver of oak savanna groundlayer plant species richness across our study region.

Local environmental conditions, specifically soil productivity, had both direct and indirect effects on groundlayer plant species richness. On more productive soils (those with positive values on soil PCA), tree canopies tended to be more closed, which decreased 1‐m^2^ groundlayer plant richness (TSE = −0.11, effect of soil productivity on richness via canopy openness). However, these effects were largely offset by a positive direct influence of soil productivity on groundlayer plant species richness (TSE = 0.23), perhaps due to greater soil resource availability on productive soils. Complex effects of soils on groundlayer plant diversity have been observed elsewhere. In longleaf pine woodlands in the southeastern United States, soil resource availability also had a positive direct effect on groundlayer richness and a negative indirect influence mediated through tree abundance (Veldman et al., 2014). In a Wisconsin oak savanna, effects of soil productivity on groundlayer richness were negligible despite negative direct effects, due to offsetting positive indirect effects via groundlayer biomass accumulation (Weiher, 2003). Together, our findings point to two key axes of resource availability that together structure groundlayer plant diversity in oak savannas: light and soil resource availability. The importance of these two resource gradients is consistent with other studies (e.g., Leach & Givnish, 1999; Peterson & Reich, 2001). Plant species richness increased with both light and soil productivity at small spatial scales in our study, but only light availability is under management control via fire and canopy thinning.

Our study did not find that thinning shrubs and saplings, either through frequent burning or mechanical treatments, promoted groundlayer plant species richness. Shrub and sapling thinning is a common management action in oak savannas, occurring at 40 of the 100 sites we sampled. However, shrub and sapling thinning may have had little influence on groundlayer plant richness because shrub layers are typically overtopped by tree canopies, meaning that management to clear shrubs and saplings may have limited effects on understory light levels under high (>80%) canopy closure. Shrub and sapling thinning is likely still an important practice in some situations, for example when invasive shrubs are present at high densities, where shrubs and saplings substantially reduce understory light availability, or where increased light availability due to fire or tree thinning results in an increase in shrub and sapling abundance (Abella et al., 2020; Taft et al., 2019; Yantes et al., 2023).

Additionally, we did not find that leaf litter accumulation, as measured by depth of litter layer, influenced groundlayer plant species richness. This result was in contrast with findings from ecologically similar longleaf pine woodlands, where high pine and broadleaf tree densities resulting from fire exclusion can decrease groundlayer plant species richness through the accumulation of duff and leaf litter (Veldman et al., 2014). We found that leaf litter accumulation increased with soil productivity but was not correlated with fire frequency, and the resulting gradient in leaf litter depth did not relate to groundlayer plant species richness. We originally hypothesized that litter depth would be most sensitive to management and important for determining groundlayer diversity. We may have found no relationship between fire frequency and litter depth because fire intensity was too low to burn through decades of leaf litter accumulation and a significant duff layer remained. Alternately, an effect of fire on leaf litter depth can be short‐lived due to rapid litter accumulation (especially if canopy density is high). Other measures of leaf litter abundance, such as biomass, percent cover, or differentiating pyrophytic oak leaf litter from litter of mesophytic trees and invasive shrubs, may reveal stronger relationships.

### The effects of management on groundlayer plant diversity vary with environmental context

We provide several lines of evidence that the effects of oak savanna management were dependent on environmental context. This context‐dependency was sometimes apparent only at a single spatial scale. At the 1‐m^2^ scale, canopy openness and species richness increased with fire frequency in less productive sites, but not in more productive sites. These findings align with our prediction that fire would more effectively open canopies and maintain openness in sandy sites, which can promote drier fuel conditions and therefore more severe fires, compared to sites with more productive soils (Leach & Givnish, 1999). Sandier soils may also have slower rates of canopy infilling following fires, allowing greater groundlayer light levels to persist and greater diversity to accumulate.

Additionally, we found that the effects of tree, shrub, and sapling thinning depended on soil conditions. Tree, shrub, and sapling thinning most effectively opened canopies at sites with unproductive soils, perhaps due to rapid growth of resprouts at sites with more productive soils (Brudvig & Asbjornsen, 2007). Furthermore, soil productivity and canopy thinning interacted to influence groundlayer richness, whereby canopy thinning was positively correlated with 1000‐m^2^ species richness at sites with high soil productivity. In contrast, there was not a corresponding increase in groundlayer richness with shrub and sapling thinning at unproductive sites, providing further evidence for the insensitivity of groundlayer richness to shrub and sapling thinning management. These results should be interpreted with some caution, as only one productive site (positive soil PCA) experienced intensive thinning (>10% of stems thinned).

Together these findings indicate the potential efficacy of varying management approaches across soil productivity gradients. Management with prescribed fire alone may be more effective at sites with less productive soils. Conversely, at sites with more productive soils, more intensive management regimes could be necessary to open canopies and promote high groundlayer plant diversity, such as the coupling of prescribed fire with tree thinning or applying fire at greater frequencies or intensities. While both fire and canopy thinning are most effective at opening the canopy in unproductive sites, canopy thinning appears to be vital for promoting groundlayer richness at more productive sites, at least early in the restoration process. As restoration progresses, fine fuels may accumulate in the groundlayer and eventually carry fires severe enough to influence canopy openness.

### The role of spatial scale for interpreting management outcomes

The restoration of groundlayer plant diversity operated differently at different spatial scales. Effects of prescribed fire and canopy thinning mediated through effects on canopy openness were apparent at the 1‐m^2^ sampling scale, but not at the 1000‐m^2^ sampling scale. We also observed a direct influence of soil productivity on plant species richness at the 1‐m^2^ sampling scale, but not at the 1000‐m^2^ sampling scale. These findings support our prediction that the roles of management and local environmental conditions on groundlayer plant diversity are scale‐dependent and indicate an important role of resource availability for the regulation of oak savanna diversity, particularly at small spatial extents. From a management perspective, had we only measured groundlayer plant species richness at the larger 1000‐m^2^ spatial scale, we may have concluded that the effects of fire and tree thinning on canopy openness had no measurable influence on groundlayer plant diversity and that overstory tree thinning had no effect on groundlayer richness whatsoever.

At small spatial extents, plant diversity can be limited by the density of groundlayer plant communities in savanna ecosystems (e.g., Veldman et al., 2014) due to relationships between abundance (the total number of individuals) and richness (the total number of species) in a local community (e.g., Storch et al., 2018). Abundance–richness relationships are mediated by resource availability (Storch et al., 2018), and our findings, along with several other studies, indicate that understory light and soil resource availability are two such key resources in oak savannas as with other ecosystems (Leach & Givnish, 1999). By this interpretation, sites with greater light availability and soil resource levels support more individual plants and therefore higher groundlayer richness. Importantly, our findings support this mechanism at only 1‐m^2^ spatial extents. This indicates greater groundlayer plant richness was a result of an accumulation of species locally, which were largely already present at the site, not from the immigration of new species to sites from the seed bank or dispersal, or prevention of site‐level extinctions. Both immigration and lack of extinctions would have also increased larger scale richness. Conversely, the fire influences on richness that were independent of canopy openness were apparent at both the 1‐m^2^ and 1000‐m^2^ scales, albeit stronger at the 1‐m^2^ scale. This indicates that there are effects of fire on richness that are unaccounted for in our analysis (e.g., other than as mediated by canopy openness or leaf litter) and that they operate at multiple scales. For example, if greater rates of flowering, seed production, and seedbank expression in response to fire increase diversity at smaller scales, these effects may contribute to diversity maintenance at larger scales over time.

### Implications for management of imperiled oak savanna ecosystems

Managers have been confronting the challenge of restoring imperiled midwestern oak savannas since at least the 1980s (Nuzzo, 1986; Packard, 1993). Here, we contextualize the results of dozens of previous studies and advance this understanding in a framework that can be used as a basis for testing additional hypotheses (Bowles & McBride, 1998; Yantes et al., 2023). By explicitly modeling both direct and indirect pathways by which management influences diversity, across broad geographic and edaphic gradients, we have combined several fundamental processes that drive oak savanna restoration outcomes. Our results emphasize the importance of light availability and soil resources for driving groundlayer plant diversity and indicate that frequent fire supports diversity by increasing light availability (among other mechanisms), especially when applied in concert with mechanical canopy thinning.

Light availability is a key resource promoting groundlayer plant diversity and is likely the resource most directly under management control. As such, it is essential to increase and maintain canopy openness, whether through prescribed fire or mechanical thinning. Restoration often proceeds in two phases facilitated by different management approaches: a “restoration” phase where management is intended to reverse decades of degradation such as fire exclusion, and a later “maintenance” phase where management more closely mimics historical natural disturbance regimes (Gann et al., 2019; Suding et al., 2004). At least in the short to medium term during the restoration phase, canopy thinning in addition to fire may be required to generate light availability that promotes sufficient fuel accumulation (e.g., herbaceous vegetation) to reach the maintenance phase where fire severity is consistently high enough to maintain canopy openness without thinning. Additional management approaches such as seed additions may facilitate the establishment of sufficient fuels, and when used in combination with canopy thinning, allow for more quickly reaching the maintenance phase (Kaul et al., 2022).

Fire promotes groundlayer richness through multiple mechanisms and has effects that vary across soil productivity gradients. Not only does prescribed fire management weakly increase richness via canopy openness (compared to the summed effect of other mechanisms), but the direct effects of fire on both richness and canopy openness appear to be more effective in low‐ compared to high‐productivity sites. The use of more intensive management regimes, like coupling burning with canopy thinning, may be important on more productive sites or sites that are less responsive to fire alone. However, our model also indicates that fire has strong direct effects on richness that are independent of its effects on canopy openness and litter depth, representing untested mechanisms. The framework we present here provides a context for testing additional hypotheses for how prescribed fire may influence diversity in ways managers can manipulate, such as via effects on nutrient cycling or germination rates, and examine their relative effect size. Incorporating different starting points for restoration could also increase the predictability of restored diversity. For example, differences in land‐use history (e.g., tillage, grazing) or duration of fire exclusion may present initial conditions that respond differently to management (Turley et al., 2020). Comparing the response of pre‐management diversity to management over time could also likely provide more nuanced insights that improve management outcomes (Bassett et al., 2020; Peterson & Reich, 2008).

The restoration of historical levels of diversity may require decades of fire and other management. Extended periods of fire exclusion and woody plant encroachment in oak savannas (i.e., several decades to more than a century) have been associated with declines in richness of over 50%, as shade‐tolerant forest species replace species with affinities for prairies and savannas (Ladwig et al., 2018). The mean richness of ~9 species/m^2^ across our study area is more consistent with richness reported from oak forests in other studies. Other savanna studies typically report richness at least twice that high (Leach & Givnish, 1999; Peterson & Reich, 2008). These savanna studies explicitly included only savanna sites with a long history of frequent fire and other management, while our study included sites at various stages in the restoration process, including unmanaged sites and those early in the process and with canopy structure intermediate between that of forests and savannas. Canopy openness in our study was 21% on average, which is on the low end of estimates from high‐quality remnant savannas that typically range from 20% to 95%. Mean fire frequency in our study is also low at ~ one burn per decade (i.e., two burns since 2000). However, these differences emphasize that both frequent fire and canopy thinning are likely needed in tandem to restore diversity in fire‐suppressed savannas. Neither approach alone is likely to restore the open canopy structure to support sufficient light availability, nor the herbaceous fuel accumulation to support sufficient fire intensity, that generates and maintains a diverse savanna groundlayer plant community.

## CONFLICT OF INTEREST STATEMENT

The authors declare no conflicts of interest.

## Supporting information


Appendix S1.

Appendix S2.

Appendix S3.

Appendix S4.

## Data Availability

Data and code (Bassett et al., 2025) are available in Dryad at https://doi.org/10.5061/dryad.wpzgmsc28.
