# Supplementary material for: A model for regional‐scale oak savanna management: The roles of fire, canopy, and soils for understory plant diversity
Source: Ecol Appl. 2025 Oct 15;35(7):e70120. doi: 10.1002/eap.70120 (PMC12524983; doi:10.1002/eap.70120)
Supplement: Supplementary file 1 — Appendix S1. [file EAP-35-e70120-s003.pdf]

Supporting Information

A regional-scale model for oak savanna management: The roles of fire, canopy, and soils for understory plant diversity

Tyler Bassett, Eric Behrens, Ralph Grundel, Johana Nifosi, Noel B. Pavlovic, and Lars A. Brudvig

Ecological Applications

Appendix S1. Oak savanna data summaries by plot cluster for environmental variables including sand content, water holding capacity, number of burns since 2000, canopy openness, litter depth, species per 1-m^2, and species per 1000-m^2 (Table S1); and principal components analysis soil parameter variables and loadings (Table S2). Includes environmental variable distributions (Figure S1) and principal components analysis graph (Figure S2).

Table S1. Data summary by plot cluster. Mean (minimum-maximum) for sand content, water holding capacity, number of burns since 2000, canopy openness, litter depth, species per 1-m^2, and species per 1000-m^2. Lat=Latitude, Long=Longitude. States: IL = Illinois, IN = Indiana, MI = Michigan, OH = Ohio, WI = Wisconsin.

Cluster	Lat	Long	# Plots	Sand Content (%)	Water Holding Capacity	# Burns Since 2000	Canopy Openness (%)
Ann Arbor	42.29	-83.73	4	57.01 (49.08-72.44)	0.69 (0.62-0.8)	2.75 (0-4)	5.41 (2.7-7.87)
ASGA North	42.6	-86.02	5	91 (89.96-91.61)	0.39 (0.43-0.45)	0.6 (0-1)	36.2 (14.35-85.85)
ASGA South	42.53	-85.96	5	91.22 (89.09-94.82)	0.44 (0.38-0.48)	0.4 (0-1)	23.37 (4.78-56.91)
Calumet	41.66	-87.01	3	90.38 (87.63-91.92)	0.42 (0.41-0.43)	0 (0-0)	15.53 (10.56-18.02)
Comstock	42.33	-85.46	3	78.06 (68.04-87.36)	0.43 (0.39-0.47)	1.67 (0-4)	15.7 (12.48-21.35)
Dexter-Huron	42.33	-83.87	2	65.78 (61.1-70.45)	0.95 (0.76-1.14)	2 (0-4)	16.04 (11.09-20.99)
FCTC	42.3	-85.32	4	75.9 (63.93-84.26)	0.53 (0.46-0.64)	3 (0-8)	7.73 (4.16-13.08)
Hayes	43.72	-85.8	3	89.92 (87.82-91.49)	0.37 (0.35-0.38)	0.33 (0-1)	43 (13.1-60.35)
Hobart	41.53	-87.3	4	26.57 (20.95-34.39)	0.67 (0.61-0.76)	2.75 (0-6)	14.46 (3.12-29.28)
Howe's Prairie	41.65	-87.07	3	96.32 (95.99-96.96)	0.39 (0.37-0.41)	3.33 (0-5)	49.1 (15.31-95.03)
Huron Meadows	42.49	-83.78	2	86.36 (84.66-88.06)	0.49 (0.44-0.54)	1 (0-2)	7.4 (4.21-10.58)
Illinois Beach	42.46	-87.8	5	93.02 (89.95-95.73)	0.47 (0.35-0.75)	1.8 (0-4)	31.59 (14.96-66.02)
Indiana Dunes State Park	41.66	-87.05	3	94.24 (94.1-94.44)	0.45 (0.43-0.47)	1.33 (0-3)	14.33 (5.56-30.11)
Inland Marsh	41.61	-87.21	3	93.8 (92.97-94.4)	0.46 (0.45-0.46)	4.33 (1-7)	25.16 (5.33-36.11)
Iron Creek	42.09	-84.1	4	71.83 (65.97-80.27)	0.42 (0.36-0.45)	3.25 (0-7)	19.67 (5.9-44.2)
Kalamazoo Moraine	42.24	-85.74	3	86.5 (81.69-89.9)	0.38 (0.37-0.39)	0.33 (0-1)	11.56 (9.1-15.24)
Kettle Moraine	42.83	-88.63	4	40.99 (28.75-59.68)	0.62 (0.57-0.69)	5.75 (0-13)	6.27 (5.49-7.9)
Kitty Todd	41.61	-83.79	7	87.91 (82.39-95.72)	0.52 (0.33-0.64)	1.14 (0-3)	27.22 (14.43-53.87)
Lake County	42.26	-87.91	3	23.02 (21.89-24.37)	0.7 (0.6-0.88)	3.33 (1-5)	9.61 (6.69-12.84)
Lowell	42.91	-85.35	3	91.5 (88.39-94.62)	0.38 (0.36-0.4)	0.33 (0-1)	20.83 (9.93-42.48)
MacCready	42.13	-84.39	3	80.25 (77.7-82.81)	0.5 (0.5-0.51)	2.33 (0-4)	10.86 (6.89-16.01)
Miller Woods	41.61	-87.26	3	91.72 (89.6-93.33)	0.55 (0.49-0.68)	3.67 (0-6)	25.1 (3.93-53.09)
Morton Arboretum	41.82	-88.06	4	19.98 (18.57-21.81)	0.68 (5.66-0.8)	4.75 (0-11)	6.8 (4-8.84)
Olson Oak Woods	42.95	-89.59	3	34.08 (13.18-71.82)	0.54 (0.45-0.6)	1.67 (0-3)	8.82 (2.29-13.34)
Otto	43.49	-86.25	5	90.46 (87.32-92.14)	0.44 (0.37-0.53)	0.4 (0-1)	39.47 (7.33-65)
Pines Point	43.52	-86.14	4	92.45 (91.97-93.03)	0.39 (0.37-0.4)	1 (0-2)	31.43 (21.71-40.46)
StoneCo Site	41.75	-83.66	2	57.41 (54.9-59.93)	1 (0.89-1.12)	0.5 (0-1)	16.6 (10.87-22.33)
Waterloo	42.35	-84.07	3	85.88 (84.08-87.83)	0.45 (0.36-0.55)	0.33 (0-1)	33 (3.78-89.37)
Overall Mean			3.57	75.09 (13.18-96.96)	0.51 (0.33-1.14)	1.93 (0-13)	21.39 (2.29-95.03)

Table S1, continued.

Cluster	# Plots	Litter Depth (cm)	# Species per 1-m²	# Species per 1000-m²	State	Ownership
Ann Arbor	4	2.08 (1.08-2.99)	8.05 (2.5-13)	50.75 (29-68)	MI	City of Ann Arbor
ASGA North	5	2.53 (1.55-3.01)	6.14 (4.1-7.8)	43.6 (29-69)	MI	MI Dept of Natural Resources
ASGA South	5	2.51 (2.13-2.69)	5.94 (4.6-7.4)	34.4 (22-52)	MI	MI Dept of Natural Resources
Calumet	3	2.54 (2.04-3.26)	5.13 (4.6-6.1)	32 (26-42)	IN	National Park Service
Comstock	3	2.06 (1.51-2.6)	7.53 (3.4-11.1)	58 (47-65)	MI	SW MI Land Conservancy
Dexter-Huron	2	2.36 (1.18-3.55)	10.65 (8.3-13)	63.5 (45-82)	MI	Huron-Clinton Metropark Authority
FCTC	4	2.62 (2.18-3.35)	8.03 (2.4-11.4)	55.5 (28-72)	MI	MI Dept of Military and Veterans Affairs
Hayes	3	3.12 (3.03-3.3)	7.67 (5-10)	37 (18-54)	MI	US Forest Service
Hobart	4	1.19 (0.72-1.63)	10.4 (5.6-15)	63.25 (53-78)	IN	National Park Service, Save the Dunes, Shirley Heinze Land Trust
Howe's Prairie	3	1.82 (0.56-3.11)	10.63 (5.7-15.3)	56.67 (45-66)	IN	National Park Service
Huron Meadows	2	2.45 (1.31-3.6)	9.65 (7.4-11.9)	70.5 (41-100)	MI	Huron-Clinton Metropark Authority
Illinois Beach	5	3.3 (3.01-3.6)	11.12 (7.4-14.9)	70 (56-76)	IL	Illinois Dept of Natural Resources
Indiana Dunes State Park	3	2.46 (1.54-3.48)	6.2 (3.3-10.5)	40.67 (26-52)	IN	IN Dept of Natural Resources
Inland Marsh	3	2.69 (2.41-3.21)	10.17 (7.3-11.8)	65 (37-85)	IN	National Park Service
Iron Creek	4	1.5 (0.9-2.27)	14.72 (5.8-26.2)	83.25 (53-113)	MI	Private
Kalamazoo Moraine	3	2.04 (1.28-2.98)	7.93 (4.3-12.3)	61.33 (42-73)	MI	SW MI Land Conservancy, Kalamazoo Valley Community College
Kettle Moraine	4	1.73 (1.3-2.33)	7.58 (4.7-11.8)	49.5 (40-58))	WI	WI Dept of Natural Resources
Kitty Todd	7	3.1 (1.15-4.46)	9.81 (7.7-14)	51 (34-107)	OH	The Nature Conservancy, Metroparks Toledo
Lake County	3	1.77 (1.18-2.39)	12.43 (2.8-19.7)	63.67 (20-92)	IL	Lake County Forest Preserve District
Lowell	3	2.51 (0.71-4.08)	8.43 (6.2-11.1)	62.67 (42-78)	MI	Land Conservancy of W MI, Lowell Twp
MacCready	3	1.32 (0.6-2.76)	11.7 (10.8-12.4)	64.33 (55-75)	MI	Michigan State University
Miller Woods	3	1.97 (1.68-2.33)	10.67 (4.7-17.3)	51.67 (33-69)	IN	National Park Service
Morton Arboretum	4	1.49 (1.06-1.77)	11.7 (4.4-16.4)	56 (43-65)	IL	Morton Arboretum
Olson Oak Woods	3	1.89 (1.21-2.25)	10.43 (4.7-14.2)	52.33 (38-62)	WI	WI Dept of Natural Resources
Otto	5	2.97 (2.4-4)	8.8 (5.3-12.3)	42.2 (21-51)	MI	US Forest Service
Pines Point	4	2.03 (0.88-3.35)	4.55 (3.1-5.7)	23.25 (17-37)	MI	US Forest Service
StoneCo Site	2	1.38 (1.16-1.59)	8.05 (5.7-10.4)	47.5 (29-66)	MI	Whiteford Twp
Waterloo	3	3.22 (0.31-5.58)	7.33 (3.8-12.5)	47.67 (36-63)	MI	MI Dept of Natural Resources
Overall Mean	3.57	2.29 (0.31-5.58)	8.94 (2.4-26.2)	53.47 (35.9-70.4)		

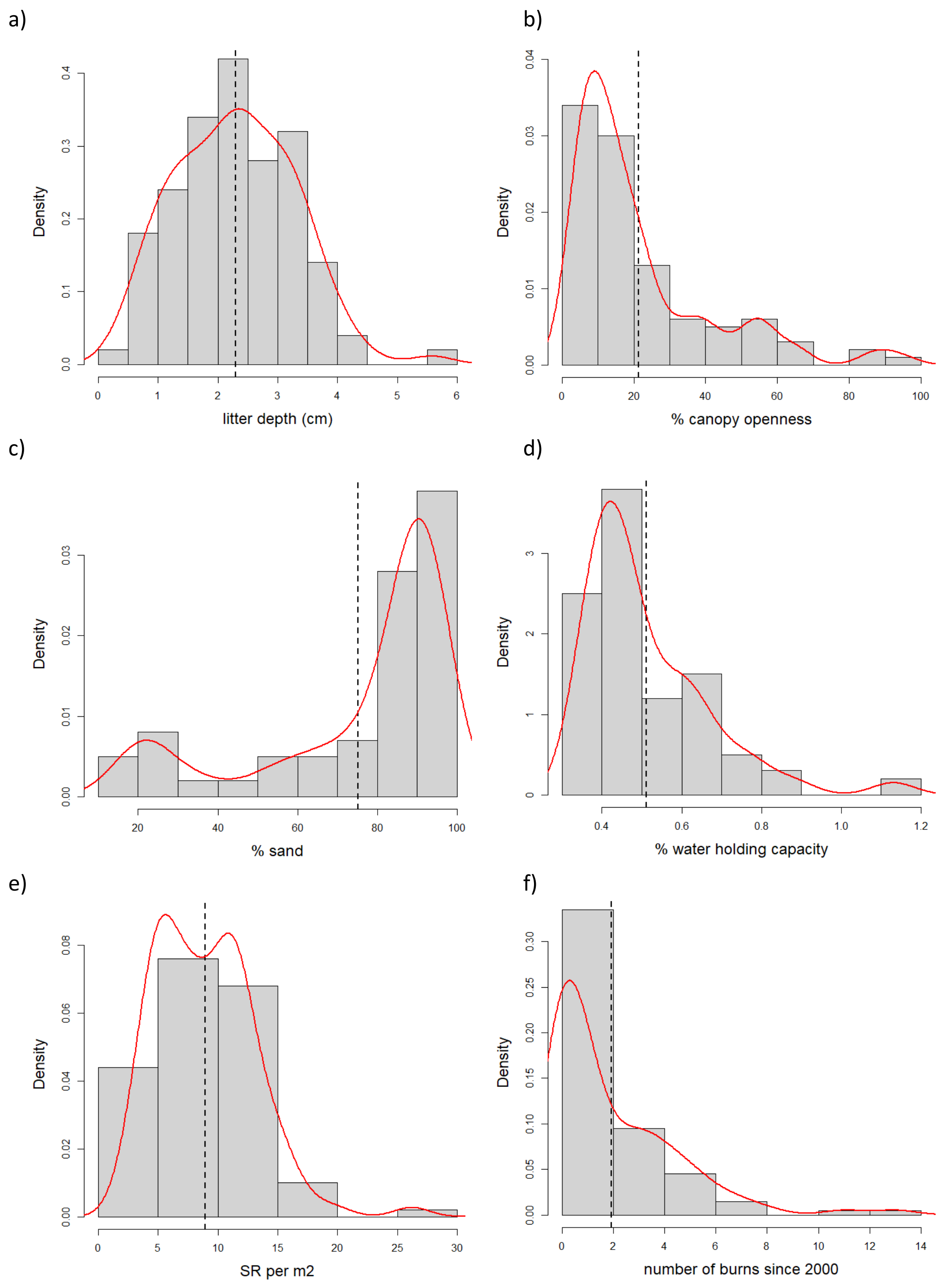

Figure S1. Distributions of litter depth (cm) (a), canopy openness (%) (b), percent sand (c), percent water holding capacity (d), species richness (SR) per m^2 (e), and number of burns since 2000 (f), summarized in Table S1. Mean shown by dashed line and red curve is continuous frequency curve.

Table S2. Summary (minimum, maximum, mean, and median) of oak savanna soil data and principal component analysis loadings for first principal components axis (PC1). The data was collected from 100 oak savanna sites across the southern Great Lakes region.

Soil variables	Minimum	Maximum	Mean	Median	PC1	PC2
Clay (%)	0.36	33.34	8.606	4.355	0.3995	0.2205
Silt (%)	0.94	63.63	16.308	8.275	0.3786	0.3075
Sand (%)	13.18	96.96	75.09	87.72	-0.392	-0.2830
Total Exchange Capacity (meq/100 g)	1.84	32.75	8.387	6.375	0.4032	-0.0205
pH	3.8	7.9	5.483	5.3	0.1919	-0.6442
Organic matter (%)	1.37	10.57	3.884	3.29	0.3844	-0.0337
Phosphorus (ppm)	1	88	20.41	12.5	-0.1901	0.5808
Nitrogen (%)	0.01	1.31	0.2232	0.145	0.1775	-0.0539
Water holding capacity (%)	0.32	1.141	0.5115	0.4565	0.3584	-0.1410

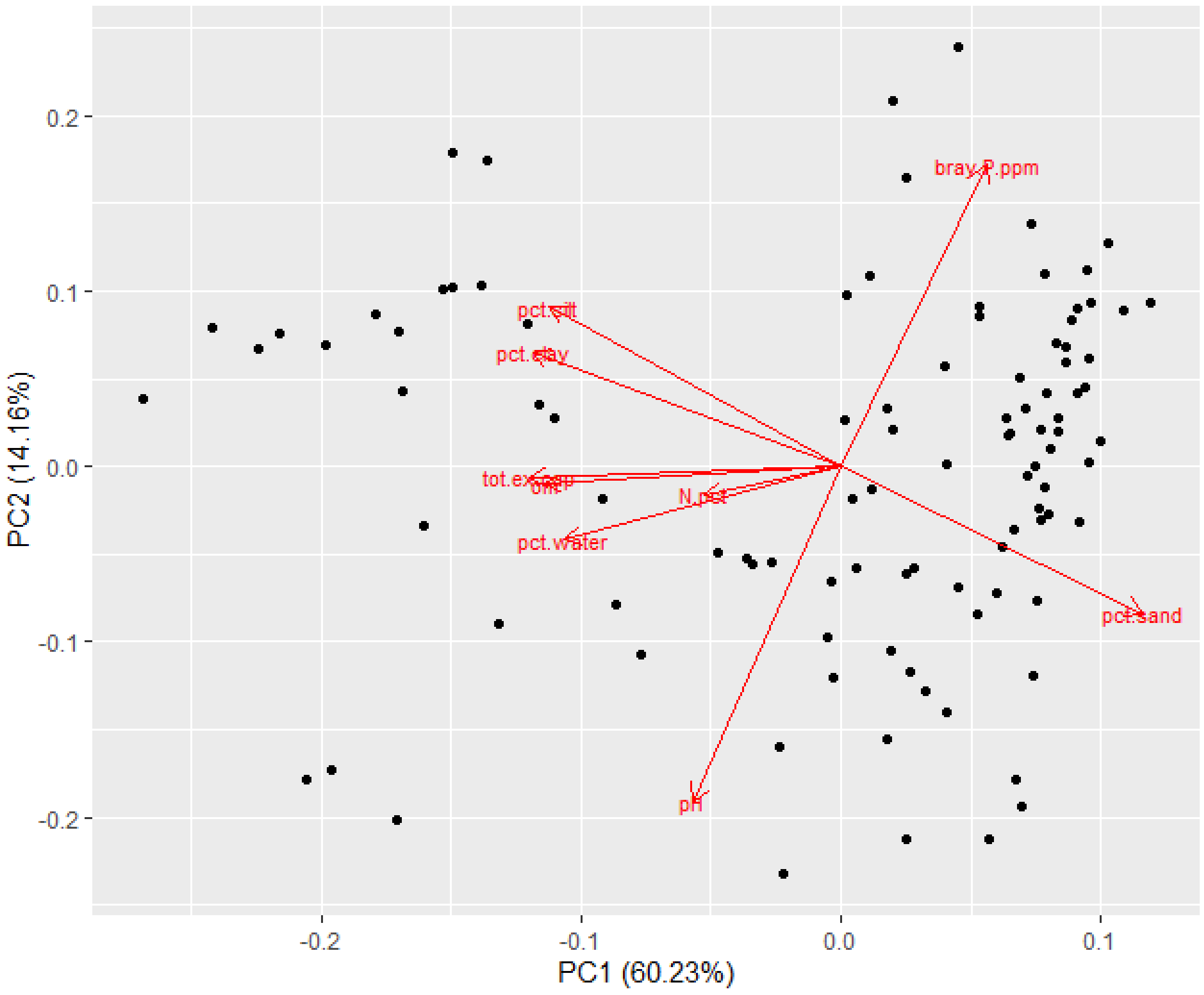

Figure S2. Bivariate plot of principle components of soils data from 100 oak savanna sites in the lower Midwest. The inverse of principle component 1 (PC1) was used in analyses, to correspond to intuitive interpretations of soil productivity. This graph depicts original principle components: % sand (pct.sand) was strongly associated with positive values of PC1; and % silt (pct.silt), % clay (pct.clay), % water holding capacity (pct.water), % nitrogen (N.pct), total exchange capacity (tot.ex.cap), and organic matter (om) was strongly associated with negative values of PC1. Percent sand was strongly positively associated with PC2 and phosphorus was strongly positively associated with PC2.
