# Supplementary material for: A model for regional‐scale oak savanna management: The roles of fire, canopy, and soils for understory plant diversity
Source: Ecol Appl. 2025 Oct 15;35(7):e70120. doi: 10.1002/eap.70120 (PMC12524983; doi:10.1002/eap.70120)
Supplement: Supplementary file 2 — Appendix S2. [file EAP-35-e70120-s004.pdf]

Supporting Information

A regional-scale model for oak savanna management: The roles of fire, canopy, and soils for understory plant diversity

Tyler Bassett, Eric Behrens, Ralph Grundel, Johana Nifosi, Noel B. Pavlovic, and Lars A. Brudvig

Ecological Applications

Appendix S2. Theoretical basis for hypothesized relationships in the structural equation model (SEM) metamodel (Figure S1).

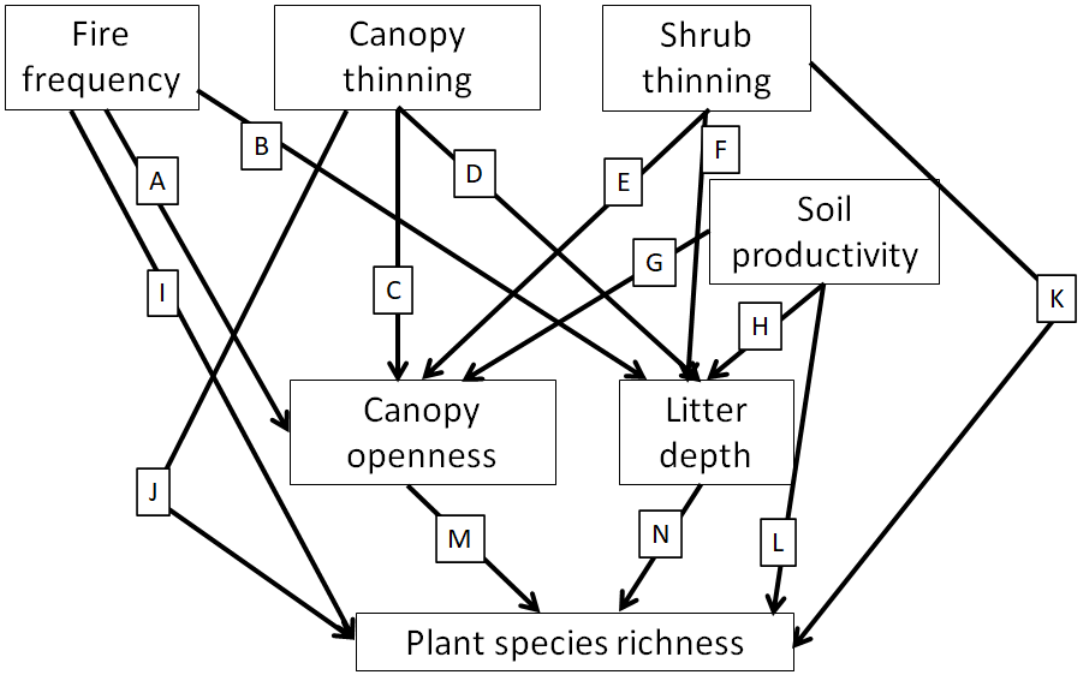

Figure S1. Diagram of the structural equation model (SEM) that was tested and evaluated with the collected oak savanna understory vegetation data (SEM) metamodel. Justification for the links (SEM paths that are identified by the alphabetic letters) are provided below.

A. Fire -> Canopy openness. Tree mortality (maybe some mid-canopy, smaller stems) increases with more frequent fire, which will increase light availability to ground. (Peterson and Reich 2001).

B. Fire -> Litter (depth, cover). More frequent fire consumes and limits accumulation of leaf litter (Veldman et al. 2013; 2014).

C. Canopy thinning -> Canopy openness. Canopy openness increases with the proportion of canopy trees cut, although this effect may be ephemeral (Brudvig and Asbjornsen 2009, Bassett et al. 2020).

D. Canopy thinning -> Litter (depth, cover). The accumulation of litter decreases with the proportion of canopy trees cut, because leaf litter deposition correlates with the number and size of canopy trees (Veldman et al. 2013, 2014).

E. Shrub thinning -> Canopy openness. Canopy openness increases with the proportion of shrubs thinned because densiometer measurements are made at breast height, although this effect may be ephemeral (Bassett et al. 2020) and the effect may be limited as some proportion of the shrub thinning effect is below/outside of the densiometer reading.

F. Shrub thinning -> Litter (depth, cover). The accumulation of litter decreases with the proportion of shrubs thinned, because leaf litter deposition correlates with the density of shrubs.

G. Soil productivity -> Canopy openness. Canopy openness decreases with soil productivity (i.e., as % sand decreases and organic matter increases, etc.) because tree growth rates are linked to soil productivity (Carmean et al. 1989).

H. Soil productivity -> Litter (depth, cover). Plant diversity increases with soil resource availability, including water and nutrients, and these effects may be largely independent of management by fire or tree thinning (Leach and Givnish 1999, Meisel et al. 2002).

I. Fire -> Plant diversity. Other mechanisms not accounted for in the model, such as scarifying seeds, chemically/heat trigger germination, fire-induced flowering, reduced competition with fire sensitive species, or other mechanisms (Pavlovic et al. 2011, Lamont et al. 2019).

J. Canopy thinning -> Plant diversity. Canopy thinning increases plant diversity though other mechanisms not accounted for in the model, such as reduced belowground competition between canopy trees and ground layer plants (Harrington et al. 2003).

K. Shrub thinning -> Plant diversity. Shrubthinning increases plant diversity though other mechanisms not accounted for in the model, such as reduced belowground competition between shrubs and ground layer plants (Harrington et al. 2003).

L. Soil productivity -> Plant diversity. Plant diversity increases with soil productivity due to promotion of forbs and total groundlayer productivity (Leach and Givnish 1999, Weiher 2003).

M. Canopy openness -> Plant diversity increases with increased canopy openness, either due to greater resource (light) availability (Weiher et al. 2003, Peterson et al. 2007) or more heterogenous resource availability (Leach and Givnish 1999).

N. Litter (depth, cover) -> Plant diversity. Diversity decreases with leaf litter as microsites for establishment decrease and competition for light (i.e., smothering from leaves) increases (Veldman et al. 2013, 2014).

Literature Cited

Bassett, T. J., D. A. Landis, and L. A. Brudvig. 2020. Effects of experimental prescribed fire and tree thinning on oak savanna understory plant communities and ecosystem structure. Forest Ecology and Management, 464: 118047.

Brudvig, L. A., and H. Asbjornsen. 2009. The removal of woody encroachment restores biophysical gradients in Midwestern oak savannas. Journal of Applied Ecology, 46(1): 231-240.

Carmean, W. H., J. T. Hahn, and R. D. Jacobs. 1989. Site index curves for forest tree species in the eastern United States. General Technical Report-North Central Forest Experiment Station, USDA Forest Service, (NC-128).

Harrington, T. B., C. M. Dagley, and M. B. Edwards. 2003. Above-and belowground competition from longleaf pine plantations limits performance of reintroduced herbaceous species. Forest Science, 49(5): 681-695.

Lamont, B. B., T. He, and Z. Yan. 2019. Evolutionary history of fire-stimulated resprouting, flowering, seed release and germination. Biological Reviews, 94(3): 903-928.

Leach, M. K., and T. J. Givnish. 1999. Gradients in the composition, structure, and diversity of remnant oak savannas in southern Wisconsin. Ecological Monographs, 69(3): 353-374.

Meisel, J., N. Trushenski, and E. Weiher. 2002. A gradient analysis of oak savanna community composition in western Wisconsin. Journal of the Torrey Botanical Society, 129: 115-124.

Pavlovic, N. B., S. A. Leicht-Young, and R. Grundel. 2011. Short-term effects of burn season on flowering phenology of savanna plants. Plant Ecology, 212(4): 611-625.

Peterson, D. W., and P. B. Reich. 2001. Prescribed fire in oak savanna: fire frequency effects on stand structure and dynamics. Ecological Applications, 11(3): 914-927.

Peterson, D. W., P. B. Reich, and K. J. Wrage. 2007. Plant functional group responses to fire frequency and tree canopy cover gradients in oak savannas and woodlands. Journal of Vegetation Science, 18(1): 3-12.

Veldman, J. W., W. B. Mattingly, and L. A. Brudvig. 2013. Understory plant communities and the functional distinction between savanna trees, forest trees, and pines. Ecology, 94(2): 424-434.

Veldman, J. W., L. A. Brudvig, E. I. Damschen, J. L. Orrock, W. B. Mattingly, and J. L. Walker. 2014. Fire frequency, agricultural history and the multivariate control of pine savanna understorey plant diversity. Journal of Vegetation Science, 25(6): 1438-1449.

Weiher, E. 2003. Species richness along multiple gradients: testing a general multivariate model in oak savannas. Oikos, 101(2): 311-316.
