# Supplementary material for: A model for regional‐scale oak savanna management: The roles of fire, canopy, and soils for understory plant diversity
Source: Ecol Appl. 2025 Oct 15;35(7):e70120. doi: 10.1002/eap.70120 (PMC12524983; doi:10.1002/eap.70120)
Supplement: Supplementary file 3 — Appendix S3. [file EAP-35-e70120-s002.pdf]

Supporting Information

A regional-scale model for oak savanna management: The roles of fire, canopy, and soils for understory plant diversity

Tyler Bassett, Eric Behrens, Ralph Grundel, Johana Nifosi, Noel B. Pavlovic, and Lars A. Brudvig

Ecological Applications

Appendix S3. Additional significant interactive effects of structural equation model (SEM) of samples of groundlayer vegetation at 100 oak savanna sites across the southern Great Lakes. Illustrated are the interactive effect of proportion canopy thinned and soil PCA gradient on canopy openness (% light) (Figure S1), effect of proportion shrub layer thinned and soil PCA gradient on canopy openness (% light) (Figure S2) and effect of proportion of the canopy thinned and soil PCA gradient on species richness (SR) per 1000 m2 (Figure S3).

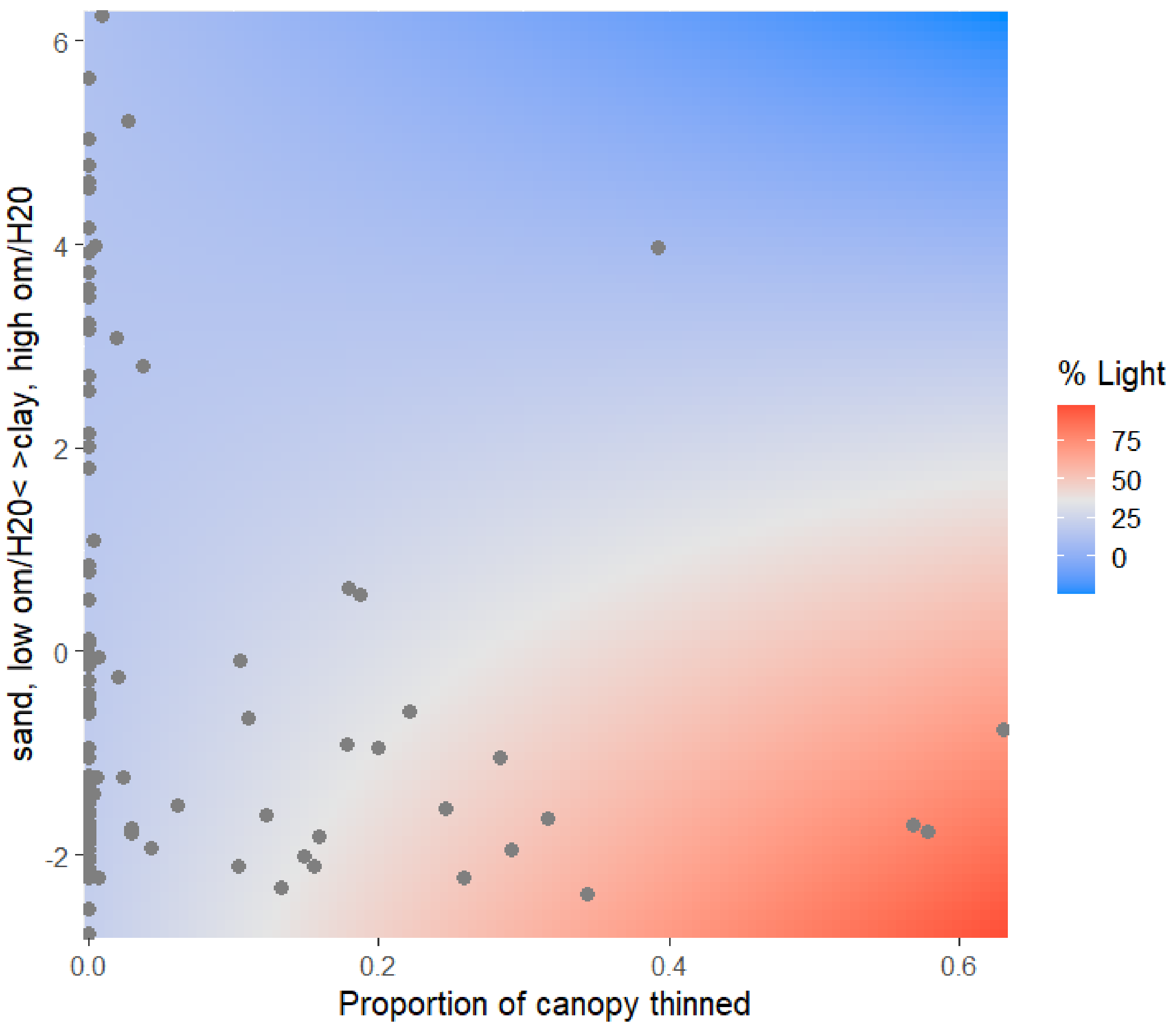

Figure S1. Canopy openness (% light) increased with proportion of canopy thinned in less productive sites (sand, low organic matter [om] and water H^{2}0]), but not in more productive sites based on groundlayer samples taken at 100 oak savanna sites in the southern Great Lakes region.

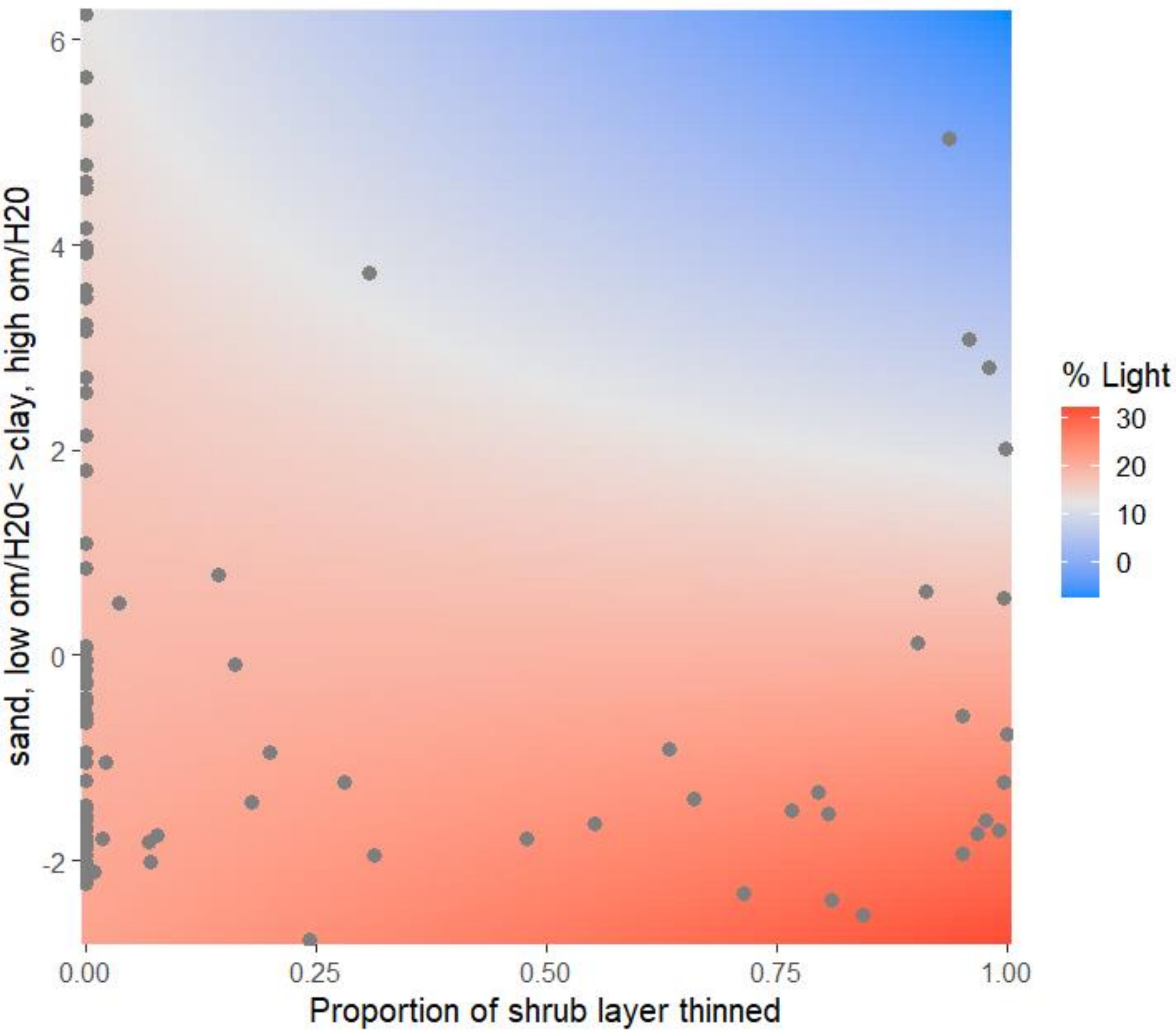

Figure S2. Canopy openness (% light) increased with proportion of shrub layer thinned in less productive sites (sand, low organic matter [om] and water H^20]), but decreased in more productive sites. Analyzed groundlayer data was from 100 oak savanna sites across the southern Great Lakes region.

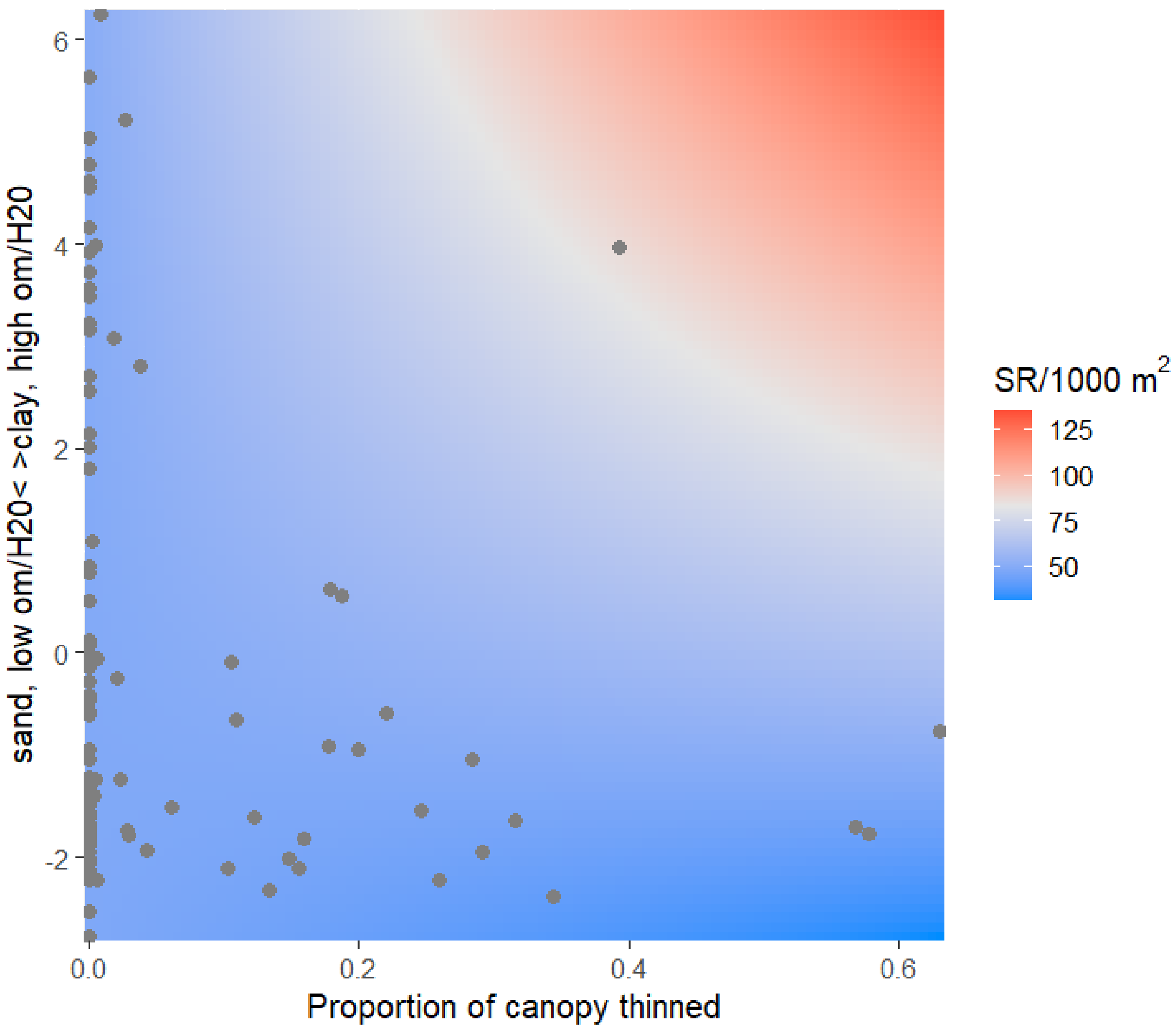

Figure S3. Species richness (SR)/1000 m^2 increased with canopy thinning in more productive sites (high clay, organic matter [om] and water (H^20)]) but not in less productive sites. Analyzed groundlayer data was from 100 oak savanna sites across the southern Great Lakes region.
