# Supplementary material for: A model for regional‐scale oak savanna management: The roles of fire, canopy, and soils for understory plant diversity
Source: Ecol Appl. 2025 Oct 15;35(7):e70120. doi: 10.1002/eap.70120 (PMC12524983; doi:10.1002/eap.70120)
Supplement: Supplementary file 4 — Appendix S4. [file EAP-35-e70120-s001.pdf]

Supporting Information

A regional-scale model for oak savanna management: The roles of fire, canopy, and soils for understory plant diversity

Tyler Bassett, Eric Behrens, Ralph Grundel, Johana Nifosi, Noel B. Pavlovic, and Lars A. Brudvig

Ecological Applications

Appendix S4. Pictures displaying differences in site conditions and management history, including comparisons of burned and unburned sites at different soil productivity (Figure S1) and comparisons of different combinations of management (fire frequency, canopy thinning intensity, shrub thinning intensity) at one cluster (Figure S2).

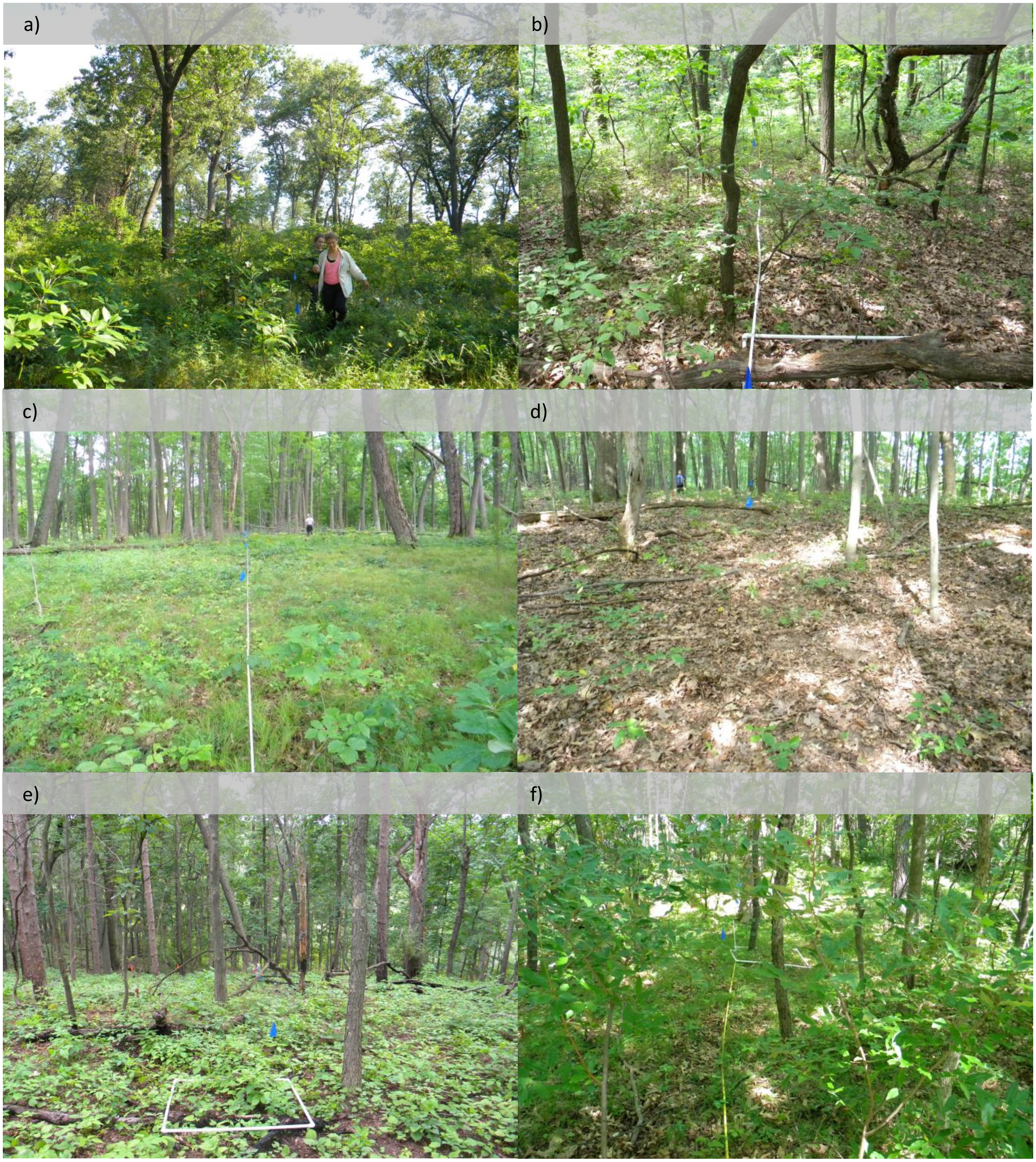

Figure S1. A comparison of treatment effects across different site conditions. *Top row*: Howe's Prairie in NW Indiana, very low soil productivity. a) Howe's North, burned 5 times since 2000; b) Howe's Woods, burned 0 times since 2000. *Middle row*: Fort Custer Training Center in SW Michigan, slightly low soil productivity. c) Area 7 Knoll, burned 8 times since 2000; d) Hart's Lake North, burned 0 times since 2000. *Bottow row*: MacCready Reserve in SE Michigan, slightly high soil productivity. e) Foxdog Hill, burned 3 times since 2000; f) MacCready Control, burned 0 times since 2000. All photos by Tyler J. Bassett.

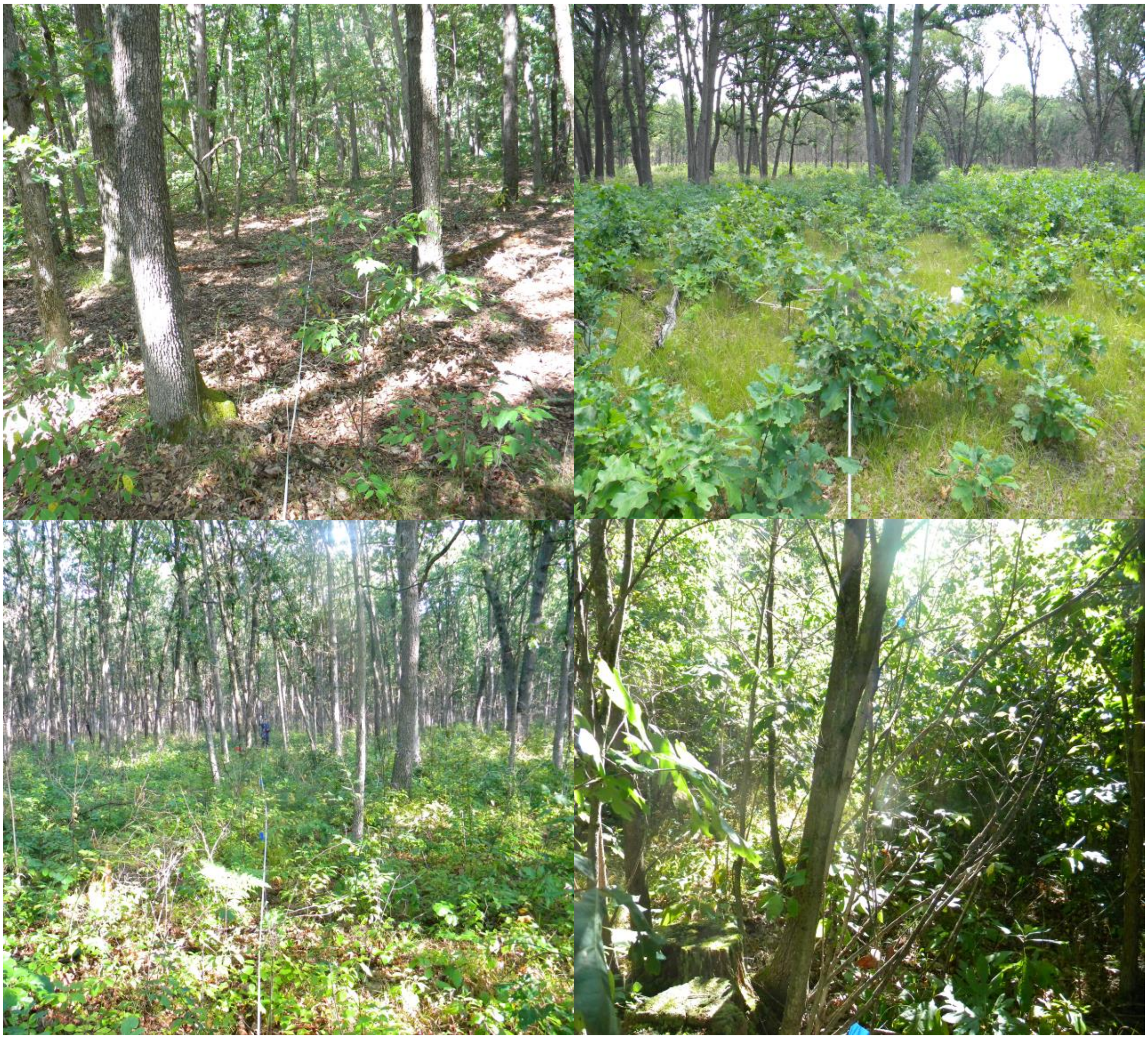

Figure S2. A comparison of treatment effects across one site, the ASGA North cluster in SW Michigan. *Clockwise from upper left*: Woods 126th, unmanaged; Barrens 126th, one prescribed fire, one canopy thinning, and two shrub thinnings since 2000; TImbercut 126th, one canopy thinning since 2000; Woods 46th, one prescribed fire since 2000. All photos by Tyler J. Bassett.
